# Data-Driven Subtyping of Parkinson’s Disease Using Longitudinal Clinical Records: A Cohort Study

**DOI:** 10.1038/s41598-018-37545-z

**Published:** 2019-01-28

**Authors:** Xi Zhang, Jingyuan Chou, Jian Liang, Cao Xiao, Yize Zhao, Harini Sarva, Claire Henchcliffe, Fei Wang

**Affiliations:** 1 Department of Healthcare Policy and Research, Weill Cornell Medical College, Cornell University, New York, USA; 2 Department of Automation, Tsinghua University, Beijing, China; 3 AI for Healthcare, IBM Research, Cambridge, USA; 4 Department of Neurology, Weill Cornell Medical College, Cornell University, New York, USA

## Abstract

Parkinson’s disease (PD) is associated with diverse clinical manifestations including motor and non-motor signs and symptoms, and emerging biomarkers. We aimed to reveal the heterogeneity of PD to define subtypes and their progression rates using an automated deep learning algorithm on the top of longitudinal clinical records. This study utilizes the data collected from the Parkinson’s Progression Markers Initiative (PPMI), which is a longitudinal cohort study of patients with newly diagnosed Parkinson’s disease. Clinical information including motor and non-motor assessments, biospecimen examinations, and neuroimaging results were used for identification of PD subtypes. A deep learning algorithm, Long-Short Term Memory (LSTM), was used to represent each patient as a multi-dimensional time series for subtype identification. Both visualization and statistical analysis were performed for analyzing the obtained PD subtypes. As a result, 466 patients with idiopathic PD were investigated and three subtypes were identified. Subtype I (Mild Baseline, Moderate Motor Progression) is comprised of 43.1% of the participants, with average age 58.79 ± 9.53 years, and was characterized by moderate functional decay in motor ability but stable cognitive ability. Subtype II (Moderate Baseline, Mild Progression) is comprised of 22.9% of the participants, with average age 61.93 ± 6.56 years, and was characterized by mild functional decay in both motor and non-motor symptoms. Subtype III (Severe Baseline, Rapid Progression) is comprised 33.9% of the patients, with average age 65.32 ± 8.86 years, and was characterized by rapid progression of both motor and non-motor symptoms. These subtypes suggest that when comprehensive clinical and biomarker data are incorporated into a deep learning algorithm, the disease progression rates do not necessarily associate with baseline severities, and the progression rate of non-motor symptoms is not necessarily correlated with the progression rate of motor symptoms.

## Introduction

Parkinson’s Disease (PD) is clinically heterogeneous, and identification of subtypes may therefore facilitate further research on underlying etiologies and development of appropriate therapies^1–3^. However, the disease is associated with a broad spectrum of variable factors including motor, cognitive, neuropsychiatric signs and symptoms, neuroimaging, genetics, and others^4^. Therefore, accurately defining PD subtypes can be challenging. Moreover, PD is a progressive neurodegenerative disorder with heterogeneity in individual disease trajectories^5^. The rationale behind this study is to utilize the comprehensive data provided by the Parkinson’s Progression Markers Initiative (PPMI)^6^ to discover PD subtypes such that the PD patients within each subtype demonstrate cohesive progression pathways. Here “pathway” refers to the longitudinal patient records and “cohesive” refers to the patient records, which are similar to each other longitudinally. We call such subtypes progression subtypes.

Robust and valuable existing studies^3,7^ on PD subtyping have defined patient groups by informative motor and non-motor variables. For instance, we can divide PD into tremor-dominant (TD) and postural instability and gait difficulty (PIGD) subtypes, according to the predefined motor criteria based upon the Unified Parkinson’s Disease Rating Scale (UPDRS). However, these conventional approaches typically just focus on one specific aspect (e.g., motor or cognition) of the patient characteristics. Therefore, we need more comprehensive approaches that can consider different aspects of patient characteristics during the subtyping process. In this case, computational techniques will likely be helpful because of the large number of variables and the complex relationships among them.

From a computational (or data-driven) perspective, patient subtyping is a clustering problem^8^, where the goal is to group patients such that each subtype corresponds to a specific patient cluster. The patients within the same subtype are therefore similar to each other. There are a small number of previous studies^1,4,9,10^ that applied data-driven clustering methodologies to identify subtypes without any prior assumptions. These methods (e.g., k-means^11,12^ or hierarchical agglomerative clustering^13^) are typically based on static patient representation derived from their baseline assessments. In this paper, we additionally incorporate longitudinal patient information into the subtyping process. This complements the subtypes identified by traditional methods as our approach can derive PD subtypes with common progression patterns.

**Figure 1:**
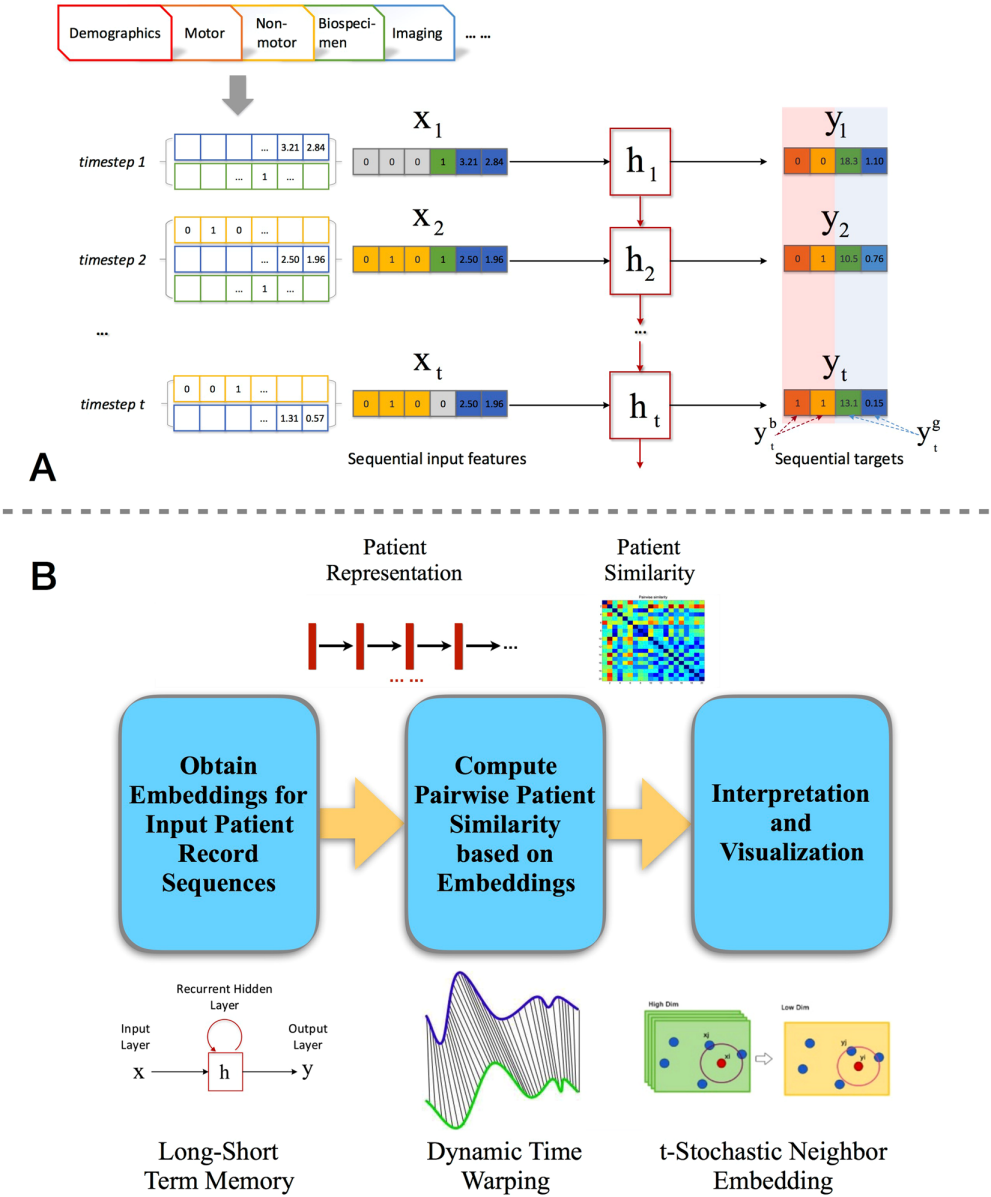
The proposed data-driven subtyping method. (**A**) Illustration of our LSTM recurrent neural network. The patient representation derived by recurrent hidden layer. Raw patient multi-source data are pre-processed by imputation. For each patient, the merged temporal records are set as input of LSTM corresponding each timestamp. The targets are a set of disjoint temporal records for each patient and obtained by the same pre-process method. There are two kinds of targets function for continuous and binary values separately. Representations generated by all the hidden states are used in patient subtyping. (**B**) Overall flow of the proposed LSTM-based method.

In order to take into account the course of PD progression, we aimed to identify progression subtypes, where the patients within each subtype are similar to each other longitudinally (in terms of the temporal trends of their records). This has the advantage of potentially providing data that could inform discussion of patient prognosis in the clinic. Therefore, quantification of the pairwise similarity between multi-dimensional longitudinal patient records would be key to discover these subtypes. To solve this problem, we first concatenated the multi-source records according to their occurring timestamps to form a temporal sequence for each patient. Then a deep learning model LSTM^14^ was trained to encode the raw record sequences into a series of standardized and dense sequence embeddings. Dynamic Time Warping (DTW)^15^, which is a common technique for quantifying the distance pairwise temporal sequences, was then applied on those embeddings to evaluate the patient similarities. Finally, the subtypes were identified through conventional clustering with the learned patient similarities (See Fig. 1).

## Methods

### Data

The patient data used in our study were obtained from the Parkinson Progression Marker Initiative (PPMI) study^6^. PPMI is an important ongoing observational, international, multi-source study that has meticulously collected various potential PD progression markers, including demographics, clinical features, imaging, and biospecimen (cerebrospinal fluid, blood, DNA, RNA) measures, that have been collected for more than six years. We downloaded the data from PPMI database on June 21, 2016. The de-identified data contained archives of enrolled subjects from June 1, 2010, to June 1, 2016. The patient features include clinical evaluation of motor and non-motor features, biospecimen examinations of cerebrospinal fluid (CSF), and neuroimaging of the dopamine transporter using ^123^I-ioflupane single photon emission computed tomography (SPECT) (DaTScan™) for this study. CSF was collected by standardized lumbar puncture procedures. Measurements of cerebrospinal fluid concentration of amyloid-beta1–42 (*Aβ*_1–42_), total Tau protein (t-Tau), and phosphorylated Tau protein at threonine 181 (p-Tau_181_) were taken in each of 102 CSF aliquots at the University of Pennsylvania using the multiplex Luminex xMAP platform (Luminex Corp). Cerebrospinal fluid alpha-synuclein concentration (*α*-syn) was analyzed at Covance using a commercially available enzyme-linked immunosorbent assay kit (Covance)^16^. DaTscan™ is a radiopharmaceutical imaging agent that works by binding to dopamine transporters (DaT) in the brain. All subjects have DAT imaging at baseline, as acquired in the striatum using SPECT. The DAT images were centrally reconstructed, attenuation corrected and analyzed with a standardized volume of interest template on caudate, putamen, and occipital regions (https://www.indd.org/).

The enrolled PD participants were required to (1) be over 30 years old; (2) have Hoehn and Yahr (H&Y) stage of PD of 1 or 2; (3) have an asymmetric resting tremor, or asymmetric bradykinesia, or two of bradykinesia, resting tremor, and rigidity with recent PD diagnosis; and (4) to be untreated by anti-PD medications^6^. Therefore, the PD patients enrolled in this study were early in their disease course, making it more likely to identify a disease progression biomarker and provide a better population for eventual disease modifying drug trials.

According to the primary diagnosis from the PPMI data, the subjects with “Idiopathic Parkinson’s Disease” or “No PD or other neurological disorder” were extracted as cases and healthy controls, respectively. In total, the dataset consisted of 15,798 records of 683 subjects including 466 PD patients. On average, each patient had approximately 23 records. We used all patients, including both cases and controls, for training LSTM based embedding, and subsequently, PD cases were used for subtyping and statistical analysis.

As there were lots of missing entries in patient records (for instance, there are 14.42% missing values for age, 15.29% missing values for disease duration), an imputation procedure with Multiple Imputation with Chained Equation (MICE)^17^ was conducted.

### PD Subtyping

We used a deep learning model for pre-processing the patient record sequences. Deep learning methods^18,19^ are normally composed of multiple layers of computational units that can perform nonlinear transformations of input features. Empirical results in certain medical applications^20,21^ have demonstrated that these learned representations often result in much improved performance compared with traditional approaches. Recently researchers have also started exploring the applications of deep learning in the tasks of learning patient representations from Electronic Health Records (EHR)^22^.

In this study, we proposed to learn patient representations with the LSTM model, which is a popular deep learning model for sequence representation learning and it has been successfully applied in tasks like speech analysis and natural language processing^14,23^. Before applying LSTM, we first concatenated patient records from different sources into an ordered sequence according to their associated timestamps as demonstrated in Fig. 1A. Moreover, we split the events in patient records (termed “features”) into two different types: input features and target features. The target features are critical variables from previous clinical studies that have been shown to be closely related to PD progression^5^. The rest of the features were treated as input features.

For each patient *p*, we used a sequence of his/her input features ${{\bf{x}}}_{t},\,t=\mathrm{1,}\,\mathrm{2,}\,\cdots ,\,{N}_{p}$ and a sequence of his/her target features ${{\bf{y}}}_{t},\,t=\mathrm{1,}\,\mathrm{2,}\,\cdots ,\,{N}_{p}$, then a novel sequential representation ${{\bf{h}}}_{t},\,t=\mathrm{1,}\,\mathrm{2,}\,\cdots ,\,{N}_{p}$ could be learned with LSTM. Each **h**_*t*_ is a dense vector with values on each dimension standardized to [−1, 1], and such vectors leverage the temporal context around timestamp *t*. Using this procedure, we could obtain integrated, standardized, and densified multi-dimensional sequential patient representations. The next step was then to evaluate pairwise similarities based on these derived representations.

Once the LSTM model was trained, the sequence of hidden layer representation ${{\bf{h}}}_{t},\,t=\mathrm{1,}\,\mathrm{2,}\,\cdots ,\,{N}_{p}$ that encode multi-source features were obtained for each patient. A sequence consists of vectors can be treated as embeddings. Those embeddings were dense and standardized (value between −1 and 1), which make it much more convenient to evaluate the patient similarities in those sequences. Of note, the problem of patient subtyping is intrinsically the problem of defining proper patient similarities. Using these similarities from patient records we have attempted to discern categories of disease progression.

Dynamic Time Warping (DTW)^15^ is a popular technique for measuring the distance (which can be regarded as dissimilarities) between pairwise temporal sequences. Different from straightforward Euclidean distance calculation, DTW first aligns the two sequences using a dynamic programming procedure and then calculates the Euclidean distance between the aligned sequences. In this way, we can consider the time shift in the evaluation process and make the results more accurate and robust. Gaussian function is employed to transform those DTW distances into similarities^24^. We evaluated such similarity for each pair of patients and form an *N* by *N* symmetric patient similarity matrix ${\bf{S}}\in {{\mathbb{R}}}^{N\times N}$. The (*i*, *j*)-th entry *S*_*ij*_ is the similarity between patient *i* and patient *j*.

Student t-Distributed Stochastic Neighbor Embedding (t-SNE)^25,26^ was adopted on to embed the patients into a 2-dimensional space so that the patient similarities could be preserved. Then the patient subtypes could be identified by performing clustering the 2-dimensional space with the k-means algorithm^11^, and the number of clusters is determined by the Hartigan’s rule^27^.

### Model Evaluation

As introduced above, our patient subtyping process includes three steps (1) representation learning with LSTM; (2) similarity calculation with DTW; (3) embedding with t-SNE and clustering with k-means. LSTM processing is a key step. To assess its effectiveness, we compared the performance of our method with the baseline procedure without LSTM processing, where the target feature sequence was used as the sequential representation for patients followed by steps 2 and 3. We also constructed another baseline with vectorized patient representations, in which each patient is represented by a vector with each dimension corresponding to the summary statistic (e.g., count for codes such as diagnosis, or average for continuous values such as laboratory test values) of a specific feature over a certain time period. The patient vectors were further processed by Principal Component Analysis (PCA)^28^ to reduce the feature dimensionality and redundancy.

In order to train an LSTM model, the data were randomly divided into training, testing and validation sets with the ratio of 6:2:2, and the three sets were non-overlapped. The sequential representations of the patients can be obtained from the hidden layers of the trained LSTM. The dimensionality of each hidden units was set as 32.

In a recent study identifying clinical subtypes of PD^5^, the overall disease severity and the global composite outcome were defined by a composition of several motor and non-motor variables including Unified Parkinson’s Disease Rating Scale (UPDRS) scores, cognitive assessment, and scales of depression and anxiety. Similar features were therefore selected as target features in our study, consisting of 82 features in total, of which 70 were continuous and 12 were binary. Those features were further integrated into 10 clinical variables shown in Table 1 of the supplemental material (e.g., the variable MoCA includes 28 features)^6^. The rest of the PPMI variables were set as input features, with 319 in total.

**Table 1:** Group characteristics of patients at the baseline in the three subtypes.

	Total (N = 466)	Subtype I (N = 201)	Subtype II (N = 107)	Subtype III (N = 158)	p-value
***Number (Percentage)***					
Male	300 (64.4%)	128 (63.6%)	63 (58.8%)	109 (68.9%)	0.5717^a^
Female	166 (35.6%)	73 (36.3%)	44 (41.1%)	49 (31.0%)	
***Mean (Standard Deviation)***					
Disease Duration	6.69 (6.71)	6.13 (6.56)	6.93 (5.80)	7.33 (7.14)	0.1784^d^
Age (years)	61.70 (9.69)	58.79 (9.53)	61.93 (9.05)	65.32 (8.86)	<0.0001^c^ (III vs II, I)
Education (years)	15.57 (3.06)	15.79 (2.75)	15.76 (3.56)	15.17 (3.03)	0.1259^c^
H&Y Stage	1.51 (0.51)	1.44 (0.5)	1.52 (0.52)	1.61 (0.5)	0.0237^a^ (III vs II, I)
MDS-UPDRS Part I	5.91 (4.35)	6.92 (4.58)	7.26 (5.26)	7.68 (4.66)	<0.0001^a^ (I vs II, III, II vs III)
MDS-UPDRS Part II	5.72 (4.16)	4.52 (3.23)	5.58 (4.41)	7.37 (4.48)	0.0040^a^ (III vs II, I)
MDS-UPDRS Part III	20.44 (9.01)	18.34 (7.9)	19.99 (9.04)	23.18 (9.92)	0.0630^a^
MDS-UPDRS Part IV	0.28 (1.07)	0.21 (0.87)	0.06 (0.34)	0.43(1.35)	0.3114^b^
MoCA	27.25 (2.33)	27.75 (2.01)	27.26 (2.42)	26.63 (2.50)	0.1931^a^ (I vs II, III, II vs III)
BJLO	24.47 (4.93)	26.43 (3.15)	20.50 (7.19)	23.53 (4.47)	<0.0001^a^ (I vs II, III, II vs III)
ESS	5.77 (3.47)	5.16 (3.06)	5.53 (3.35)	6.64 (3.82)	0.2341^a^
RBD^#^	3.22 (2.66)	3.21 (2.62)	3.23 (2.49)	4.97 (3.31)	0.0009^a^ (III vs II, I)
GDS	5.26 (1.43)	5.11 (1.43)	5.2 (1.17)	5.47 (1.50)	0.2173^a^
HVLT	24.42 (4.95)	26.55 (4.14)	24.19 (5.09)	24.0 (4.97)	<0.0001^a^ (I vs II, III, II vs III)
LNS	10.70 (2.66)	11.51 (2.58)	10.52 (2.51)	9.75 (2.49)	0.0084^a^ (I vs II, III)
QUIP	0.13 (0.40)	0.15 (0.47)	0.14 (0.43)	0.18 (0.5)	0.2548^a^
SCOPA-AUT	10.52 (6.44)	8.44 (4.69)	8.85 (6.14)	13.85 (7.08)	0.0015^a^ (III vs II, I)
Semantic Fluency	48.84 (11.73)	52.75 (11.03)	48.95 (9.65)	43.81 (11.41)	0.0104^a^ (I vs II, III, II vs III)
STAI	65.30 (18.28)	61.84 (15.85)	62.14 (17.96)	71.0 (19.81)	0.4960^a^
SDMT	41.22 (9.71)	44.83(8.70)	42.48 (6.89)	36.12 (9.65)	0.0178^a^ (III vs II, I)
UPSIT	22.20 (8.11)	24.27 (7.68)	24.85 (7.07)	18.54 (7.68)	0.0029^a^ (III vs II, I)
Genetic Risk Score	−0.016 (0.01)	−0.01 (0.01)	−0.02 (0.01)	−0.02 (0.01)	0.0118^c^ (I vs III)
MCI^#^	0.15 (0.36)	0.10 (0.29)	0.09 (0.28)	0.24 (0.43)	0.0031^a^ (III vs II, I)
DaTScan Caudate SBR^#^	1.96 (0.59)	2.03 (0.56)	2.19 (0.64)	1.74 (0.55)	<0.0001^d^ (I vs II, III, II vs III)
DaTScan Putamen SBR^#^	0.82 (0.37)	0.83 (0.34)	1.02 (0.48)	0.70 (0.3)	<0.0001^d^ (I vs II, III, II vs III)
Medication Use^#^	0.00 (0.00)	0.00 (0.00)	0.00 (0.00)	0.00 (0.00)	1

^a^Chi-square test; ^b^Fisher exact test; ^c^One-way ANOVA test; ^d^Kruskal-Wallis H-test. The specific different subtypes Determined by use of Tukey post hoc analysis. Abbreviations: H&Y: Hoehn and Yahr; MDS-UPDRS: Movement Disorders Society–revised Unified Parkinson’s Disease Rating Scale; MoCA: Montreal Cognitive Assessment; BJLO: Benton Judgment of Line Orientation; ESS: Epworth Sleepiness Scale; RBD: Rapid eye movement sleep Behavior Disorder; GDS: Geriatric Depression Scale; HVLT: Hopkin’s Verbal Learning Test; LNS: Letter Number Sequencing; QUIP: Questionnaire for Impulsive-Compulsive Disorders in Parkinson’s Disease; SCOPA-AUT: SCales for Outcomes in PArkinson’s disease-AUTomotic symptoms; STAI: State Trait Anxiety Inventory; SDMT: Symbol Digit Modalities Test; UPSIT: University of Pennsylvania Smell Identification Test; MCI: Mild Cognitive Impairment; DaTScan SBR: DaTScan Striatal Binding Ratio. ^#^RBD’s rating scale is 0–10; MCI was determined by patients with cognititive declines, no functional impairment, and values of cognitive tests HVLT, BJLO, LNS, Semantic Fluency and SDMT; DaTScan SBR is calculated as (target region/reference region)−1; Medication Use defined by 0 = Unmedicated for PD, 1 = Levadopa, 2 = Dopamine Agonist, 3 = Other, 4 = Levadopa & Other, 5 = Levadopa & Dopamine Agonist, 6 = Dopamine Agonist & Other, 7 = Levadopa & Dopamine Agonist & Other.

To evaluate the effectiveness of the learned patient representation and similarities, we visualized their embeddings with t-SNE and colored the detected subtypes. We also conducted statistical analysis to identify the distinct features for different subtypes for interpretation purpose. More concretely, we used Chi-square test for the categorical variables, one-way ANOVA for the normal continuous variables, Kruskal-Wallis test for the non-normal continuous variables, and Fisher’s exact test for the high sparsity variables. For the tests with significant p-value, Tukey post hoc analysis were performed on every two subtypes to identify specific difference. Based on prior studies^5,29^, if the p-value was smaller than 0.05, we considered a significant group effect for the associated variables.

## Results

### Visualization of patient subtypes

**Figure 2:**
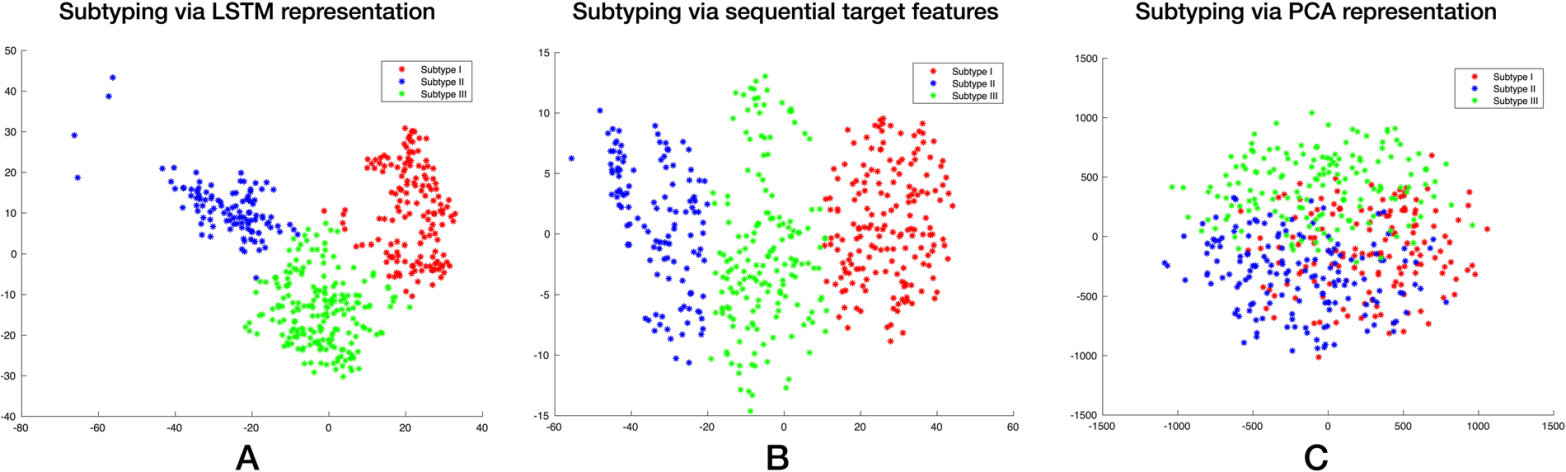
Visualization of patient subtyping results by various methods. (**A**) Representation learned by LSTM. (**B**) Dynamic time warping using sequential target features. (**C**) Representation learned using PCA with merging sequential data into static vectors. Patients are mapped to the 2-dimensional space using the t-SNE with learned representations as input. Points with three different color represent three subtypes of patients.

Figure 2 demonstrates the subtyping results with LSTM representation and the two baselines. The first one directly calculated the patient similarities with DTW on the raw target feature sequence. The second one collapses all features into a vector for each patient and then performed PCA on top of the patient vectors. Compared with Fig. 2(B,C), the three subtypes depicted by learned LSTM representation in Fig. 2(A) are much more salient, with a better separation in scatterplot (smaller intra-cluster distance and larger inter-cluster distance).

### Subtype characteristics

Patient characteristics for each subtype in Fig. 2(A), including demographics, clinical features, imaging, and biospecimen, are summarized in Tables 1 and 2 (baseline and last records). The variables contain disease duration, age, education, medication use, clinical severity measures such as H&Y Stage, MDS-UPDRS (Movement Disorders Society–revised Unified Parkinson’s Disease Rating Scale) Part I-IV, non-motor measures including cognitive impairment, depression, anxiety, sleep disorders, and imaging assessments including DaTScan Striatal Binding Ratio (SBR), as well as key CSF biomarkers (the online implementation of Subtype Characteristics Analysis is provided on https://github.com/sheryl-ai/Subtype-Analysis).

**Table 2:** Group characteristics of patients at their last records in the three subtypes.

	Total (N = 466)	Subtype I (N = 201)	Subtype II (N = 107)	Subtype III (N = 158)	p-value
***Number(Percentage)***					
Male	300 (64.4%)	128 (63.6%)	63 (58.8%)	109 (68.9%)	0.5717^a^
Female	166 (35.6%)	73 (36.3%)	44 (41.1%)	49 (31.0%)	
***Mean (Standard Deviation)***					
Disease Duration^*^	10.39 (6.93)	10.00 (6.72)	9.61 (6.34)	11.18 (7.33)	0.3184^d^
Age (years)^*^	65.39 (9.71)	62.66 (9.55)	64.61 (9.20)	69.16 (8.82)	<0.0001^c^ (III vs II, I)
Education (years)	15.57 (3.06)	15.79 (2.75)	15.76 (3.56)	15.17 (3.03)	0.1259^c^
H&Y Stage	1.89 (0.59)	1.81 (0.48)	1.66 (0.51)	2.15 (0.68)	<0.0001^a^ (III vs II, I)
MDS-UPDRS Part I	8.73 (5.66)	6.92 (4.58)	7.26 (5.26)	12.03 (5.73)	<0.0001^a^ (III vs II, I)
MDS-UPDRS Part II	9.41 (6.35)	7.48 (4.85)	6.85 (4.41)	13.61 (7.02)	<0.0001^a^ (III vs II, I)
MDS-UPDRS Part III	25.39 (12.79)	22.39 (11.86)	23.18 (9.92)	30.71 (13.93)	0.1146^a^
MDS-UPDRS Part IV	1.44 (2.57)	1.26 (2.35)	0.39 (1.22)	1.89 (2.93)	0.8847^b^
MoCA	26.64 (3.26)	27.98 (1.86)	27.09 (2.4)	24.62 (4.06)	<0.0001^a^ (I vs II, III, II vs III)
BJLO	24.47 (5.13)	26.58 (3.34)	20.64 (7.10)	23.29 (4.83)	<0.0001^a^ (I vs II, III, II vs III)
ESS	7.44 (4.62)	6.47 (3.95)	6.38 (4.06)	9.13 (5.13)	0.0300^a^ (III vs II, I)
RBD^#^	3.87 (3.00)	3.21 (2.62)	3.23 (2.49)	4.97 (3.31)	0.0036a(III vs II, I)
GDS	5.50 (1.54)	5.20 (1.31)	5.31 (1.28)	5.96 (1.80)	0.0017^a^ (III vs II, I)
HVLT	24.19 (5.99)	27.15 (4.77)	24.00 (4.97)	20.50 (5.70)	<0.0001^a^ (I vs II, III, II vs III)
LNS	10.21 (3.00)	11.43 (2.43)	10.27 (2.41)	8.64 (3.14)	<0.0001^a^ (I vs II, III, II vs III)
QUIP	0.16 (0.47)	0.15 (0.47)	0.14 (0.43)	0.18 (0.50)	0.9693^a^
SCOPA-AUT	13.64 (7.42)	11.45 (6.07)	10.02 (5.81)	17.92 (7.59)	<0.0001^a^ (III vs II, I)
Semantic Fluency	47.96 (12.34)	52.97 (10.82)	48.76 (11.02)	41.29 (11.53)	0.0609^a^
STAI	65.37 (19.27)	59.52 (16.07)	61.89 (18.15)	74.25 (20.12)	0.0053^a^ (III vs II, I)
SDMT	38.96 (12.28)	44.95 (9.98)	39.50 (7.53)	31.13 (12.09)	0.0001^a^ (I vs II, III, II vs III)
MCI^#^	0.23 (0.42)	0.16 (0.37)	0.11 (0.31)	0.37 (0.48)	<0.0001^a^ (III vs II, I)
CSF t-Tau^#^	44.68 (18.18)	41.91 (15.53)	48.72 (18.79)	46.7 (20.43)	0.0096^c^ (I vs II, III)
CSF *Aβ*_1–42_^#^	371.31 (100.68)	376.11 (97.44)	401.10 (81.15)	353.38 (108.02)	0.0052^c^ (II vs III)
CSF p-Tau_181_^#^	15.76 (10.15)	16.04 (11.53)	17.36 (9.84)	14.78 (8.04)	0.2498^d^
CSF *α*-synuclein^#^	1852.2 (792.9)	1818.9 (777.5)	2025.1 (806.1)	1828.4 (799.2)	0.1407^d^
DaTScan Caudate SBR^#^	1.71 (0.59)	1.78 (0.55)	2.08 (0.62)	1.41 (0.53)	<0.0001^d^ (I vs II, III, II vs III)
DaTScan Putamen SBR^#^	0.69 (0.35)	0.68 (0.25)	0.97 (0.48)	0.54 (0.24)	<0.0001^d^ (I vs II, III, II vs III)
Medication Use^#^	2.48 (2.24)	3.40 (2.14)	0.51 (1.21)	2.65 (2.05)	<0.0001^a^ (I vs II, III, II vs III)

^*^Disease duration and Age are calculated based on the last time points of MDS-UPDRS. ^a^Chi-square test; ^b^Fisher exact test; ^c^One-way ANOVA test; ^d^Kruskal-Wallis H-test. The specific different subtypes Determined by use of Tukey post hoc analysis. Abbreviations: H&Y: Hoehn and Yahr; MDS-UPDRS: Movement Disorders Society–revised Unified Parkinson’s Disease Rating Scale; MoCA: Montreal Cognitive Assessment; BJLO: Benton Judgment of Line Orientation; ESS: Epworth Sleepiness Scale; RBD: Rapid eye movement sleep Behavior Disorder; GDS: Geriatric Depression Scale; HVLT: Hopkin’s Verbal Learning Test; LNS: Letter Number Sequencing; QUIP: Questionnaire for Impulsive-Compulsive Disorders in Parkinson’s Disease; SCOPA-AUT: SCales for Outcomes in PArkinson’s disease-AUTomotic symptoms; STAI: State Trait Anxiety Inventory; SDMT: Symbol Digit Modalities Test; MCI: Mild Cognitive Impairment; CSF: Cerebrospinal fluid; DaTScan SBR: DaTScan Striatal Binding Ratio. ^#^RBD’s rating scale is 0–10; MCI was determined by patients with cognititive declines, no functional impairment, and values of cognitive tests HVLT, BJLO, LNS, Semantic Fluency and SDMT; CSF biomarkers’ unit is pg/ml; DaTScan SBR is calculated as (target region/reference region)-1; Medication Use defined by 0 = Unmedicated for PD, 1 = Levadopa, 2 = Dopamine Agonist, 3 = Other, 4 = Levadopa & Other, 5 = Levadopa & Dopamine Agonist, 6 = Dopamine Agonist & Other, 7 = Levadopa & Dopamine Agonist & Other.

The differences in mean age at baseline for the three patient subtypes are significant, at 58.79 ± 9.53, 61.39 ± 6.56, and 65.32 ± 8.86 years respectively. We therefore performed further multivariate analysis with adjustment to investigate the contribution of the onset-age presented in Supplement Tables 5–10. This, importantly, demonstrated no significant effect of Age after adjusting for multiple comparisons (p > 0.05). Tables 1 and 2 demonstrates important variables that were contributory in characterizing subtypes: severity rating assessed by H&Y stage, motor and non-motor assessment for MDS-UPDRS, global cognitive function assessed by Montreal Cognitive Assessment (MoCA), visuospatial abilities assessed by Benton Judgment of Line Orientation (BJLO), daytime sleepiness assessed by Epworth Sleepiness Scale (ESS), executive function/working memory assessed by Letter Number Sequencing (LNS), verbal memory assessed by Hopkin’s Verbal Learning Test (HVLT), sleep behavior assessed by Rapid Eye Movement sleep behavior disorder (RBD), depression degree assessed by Geriatric Depression Scale (GDS), impulsive-compulsive disorders assessed by Questionnaire for Impulsive-Compulsive Disorders (QUIP), autonomic dysfunction assessed by Scales for Outcomes in Parkinson’s disease-Autonomic symptoms (SCOPA-AUT), semantic testing for semantic fluency, anxiety degree assessed by State Trait Anxiety Inventory (STAI), processing speed/attention assessed by Symbol Digit Modalities Test (SDMT), olfaction measured by University of Pennsylvania Smell Identification Test (UPSIT), cognitive impairment assessed by Mild Cognitive Impairment (MCI), quantified *α*-syn, *Aβ*_1–42_, t-Tau, and p-Tau_181_ for CSF biomarkers, and DaTScan Striatal Binding Ratios (calculated by (striatal region)/(occipital) −1 from 4 h post-injection 123-I Ioflupane image)^6^. The specific mean values indicate the severity of the significant manifesting variables on the corresponding subtype.

The first subtype (Subtype I) comprised 201 patients, and was characterized by mild H&Y stage (mean value 1.81), mild non-motor symptoms (cognitive impairment, depression, anxiety) as reported by patients on MDS-UPDRS Part I, and significantly lower CSF t-Tau level. The motor severity of the second subtype (Subtype II) (107 patients) was similar to Subtype I. However, several measures of non-motor features such as MoCA, GDS, and STAI of Subtype II were more severe than in Subtype I. Of note, Subtype II had the highest CSF *Aβ*_1–42_ concentration, but the lowest BJLO (Benton Judgment of Line Orientation test) and SCOPA-AUT in independent non-motor domains. Subtype III (158 patients) has the most severe motor and non-motor symptoms.

**Figure 3:**
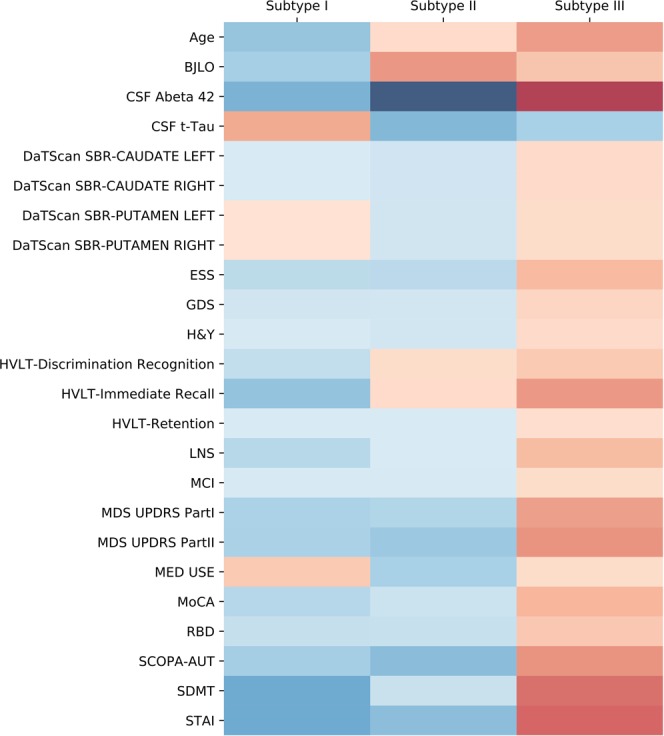
Heatmap illustration of the patient subtyping results of LSTM for three subtypes. The colors are generated according to mean values of subtypes. The red color depicts more a severe deficit for the variable and the blue color means that the symptom is less severe. The darker color represents larger difference with total average. Variables with p-value < 0.05 are shown.

We also demonstrated the discriminative power of these features though the differences between their mean values within each subtype and their global mean values, using a heatmap presented in Fig. 3. Each column in the figure represents a subtype while each row represents a feature p-value < 0.05 in the statistical testing. By comparing the profiles of the subtypes, we can see that the third subtype was older and had more severe motor and non-motor features. The first and second subtypes significantly differed by cognitive factors including MoCA, BJLO, HVLT, LNS, and SDMT, CSF biomarkers (t-Tau), as well as DaTScan SBR (the detailed mean values of these features are shown in Tables 1 and 2).

### Disease progression patterns in different subtypes

**Figure 4:**
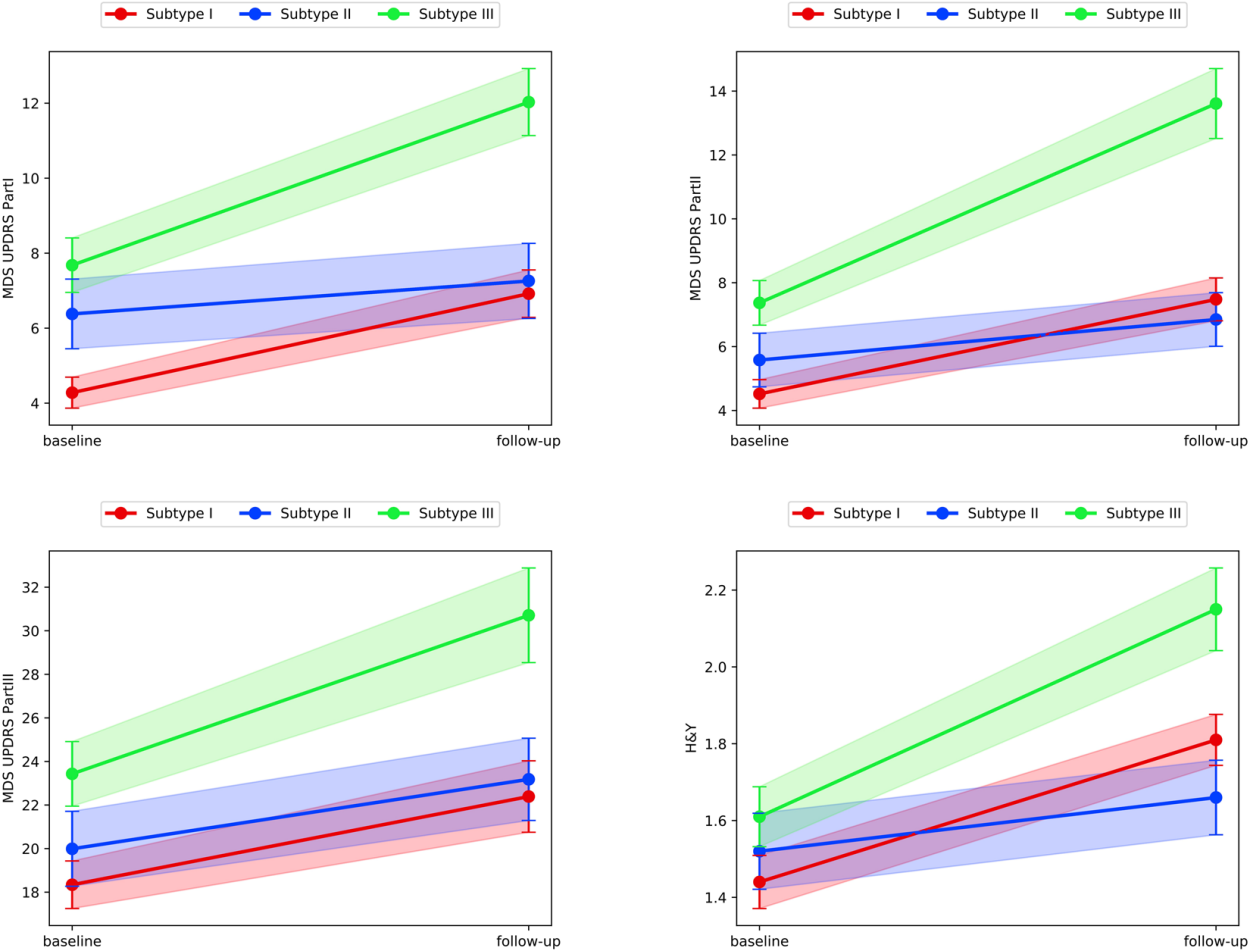
Comparisons of three Subtypes on disease progression of the variable MDS-UPDRS Part I-III, and H&Y. The time interval between baseline and follow-up is 6 years. The larger slope illustrates a more rapid progression on the corresponding variables. The filled regions indicate 95% Confidence Intervals as error bars for corresponding point estimates. Variables with the p-value < 0.05 are shown.

**Figure 5:**
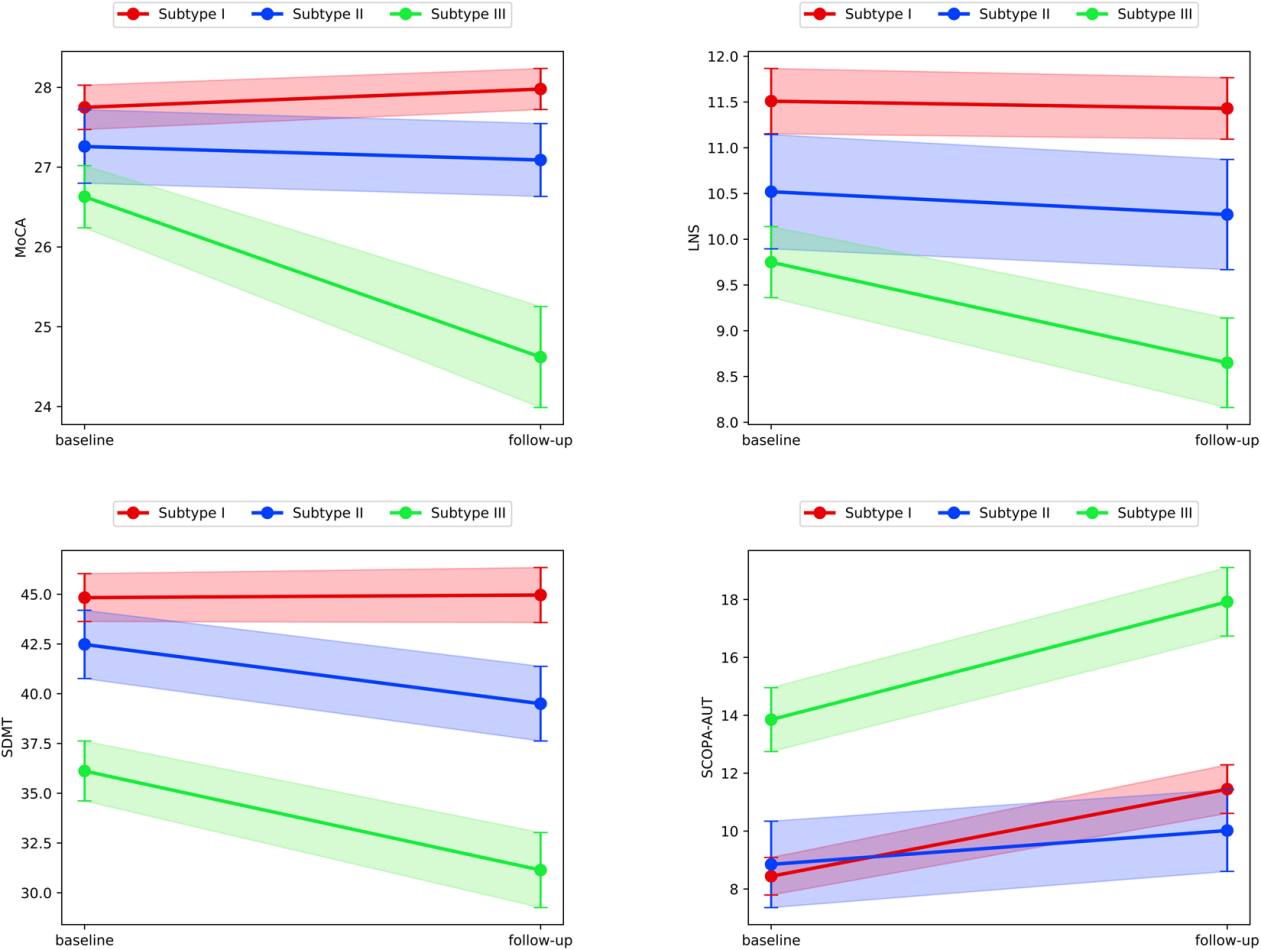
Comparisons of three Subtypes on disease progression of the variable MoCA, LNS, SDMT, and SCOPA-AUT. The time interval between baseline and follow-up is 6 years. The larger slope illustrates a more rapid progression on the corresponding variables. The filled regions indicate 95% Confidence Intervals as error bars for corresponding point estimates. Variables with the p-value < 0.05 are shown.

The existence of PPMI study follow-up data allowed us to examine disease progression patterns for different subtypes. To identify the features whose value changes are significant from baseline to follow-up visits, we conducted statistical testing on their value differences between the two visits. The variables whose value changes were significantly distinct across the three subtypes are shown in Figs 4 and 5, where greater slope indicates a more rapid progression of the specific variable in the subtype, whereas the smaller slope represents a relatively more stable condition. Based on MDS-UPDRS motor and non-motor subscores and H&Y stage, the disease progression of Subtype III is faster than Subtype I and Subtype II, while progression in Subtype II was slower than Subtype I. Non-motor measures of MoCA, LNS (Letter-Number Sequencing), SDMT (Symbol-Digit Modalities Test), and SCOPA-AUT suggested that Subtype III has the most prominent decline in general cognitive ability and autonomic function. In contrast, the cognitive abilities are relatively unchanged for Subtype I and slightly decreases for Subtype II. Subtype I had faster autonomic function progression of a compared with Subtype II. The DaTScan imaging results (See Supplement Fig. 3) of the region of interest (Caudate and Putamen) for the three subtypes suggested that the DaTScan SBR value of Subtype III decreases more significantly, which was consistent with the fact that Subtype III was associated with the most severe disease course^30^.

### Comparison with other methods

Characteristics of the subtypes at the subjects’ last study records and progression obtained through four different methods are listed (see Supplement Table 2). We analyzed all the baseline methods by statistical testing (Chi-square test; Fisher exact test; One-way ANOVA test; Kruskal-Wallis H-test) and computed p-value for each variable. For a fair comparison, DTW, t-SNE and k-means were utilized on all the subtyping methods.

Supplement Table 2 demonstrates that the variables with more markers in the column were indicative of the more sensitive variables that can be used to interpret the subtyping results. We can observe that the proposed method can identify more significant variables than the baselines, which led to more distinct patient subtypes.

## Discussion

### Clinical Interpretation of the Identified Subtypes

In this study we have identified three novel PD subtypes based upon incorporation of comprehensive clinical and biomarker data and have summarized their clinical characteristics. Specifically, we can interpret the three subtypes as follows (and we interpret the three subtypes from a more abstract perspective in Supplemental Material Table 4).

#### *Subtype I* (Mild Baseline, Moderate Motor Progression)

The patients in this subtype start with a relatively mild deficits on both their motor and non-motor capabilities at baseline. However, their motor functionalities will decay at a moderate rate over time while their cognitive abilities are relatively stable.

#### *Subtype II* (Moderate Baseline, Mild Progression)

The patients in this subtype begin with moderate deficits in both their motor and non-motor capabilities at baseline (i.e., more severe than Subtype I). Both their motor and non-motor functionalities progress slowly over time.

#### *Subtype III* (Severe Baseline, Rapid Progression)

The patients in this subtypebegin with more significant deficits in both their motor and non-motor capabilities at baseline (i.e., more severe than Subtype I and II). Both their motor and non-motor functionalities progress rapidly over time.

These analyses therefore demonstrate heterogeneity of PD progression between patient subtypes and also between classes of symptoms. From Subtype I to Subtype III, overall the subjects motor and non-motor symptoms are more severe at baseline. In particular we identify a subset of individuals with PD (Subtype III) with more severe motor and non-motor symptoms and faster disease progression rate. However, more severe onset status in our model does not necessarily lead to faster progression, since motor symptom decay rate for Subtype II is slower than Subtype I. Our analyses also suggest dissociation between the progression of non-motor symptoms and motor symptoms in specific subtypes.

Clinical experience with PD has underlined that with progression of motor symptoms, non-motor symptoms commonly worsen. However, our data support that by searching for subtypes based upon phenotypic and possibly biomarker characteristics, it may be possible to dissect out groups of individuals in whom severity of motor and non-motor symptoms does not correlate strongly. Indeed, there is already abundant evidence that non-motor symptoms may associate differentially with “traditional” clinically-based subtypes of tremor-predominant versus PIGD PD^31^. In the clinic, a more nuanced appreciation of the likely future course of a patient with PD could be highly impactful.

### Relationship with conventional PD subtypes

**Figure 6:**
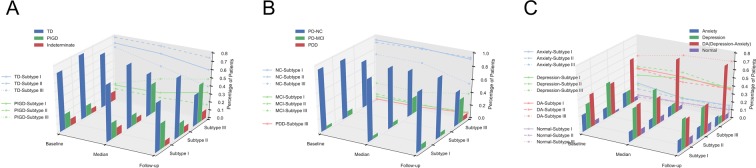
Relationship with Conventional PD Subtypes. (**A**) Patient correlation of three subtypes and motor subtypes at baseline, median time point, and 6-year follow-up. Patients are categorized into motor subtypes including TD, PIGD, and Indeterminate. The longitudinal correlation of three subtypes with TD and PIGD are plotted by lines respectively. (**B**) Patient correlation of three subtypes and cognitive subtypes at baseline, median time point, and 6-year follow-up. Patients are categorized into cognitive subtypes including Normal Cognition (PD-NC), Mild Cognitive Impairment (PD-MCI), and Dementia (PDD). The longitudinal correlation of three subtypes with PD-NC, PD-MCI, and PDD are plotted by lines respectively. (**C**) Patient correlation of three subtypes and mood subtypes at baseline, median time point, and 6-year follow-up. Patients are categorized into mood subtypes including Anxiety, Depression, Depression-Anxiety (DA), and Normal Mood. The longitudinal correlation of three subtypes with Anxiety, Depression, and Anxiety-Depression are plotted by lines respectively.

Conventionally there are two well-described motor PD subtypes based upon UPDRS scores, (1) Tremor-Dominant PD (TD); and (2) Postural Instability and Gait Difficulty (PIGD)^7^. In PPMI, the motor subtypes can be defined based on MDS-UPDRS^32^: cutoff scores of $\leqslant$1.15 for TD classification and $\geqslant$0.90 for PIGD; if the ratio is between the cutoff scores 0.90 and 1.15, then the patient is classified as indeterminate. We therefore examined prevalence of TD and PIGD in our subtypes at different time points (Fig. 6A). For Subtype I and II, more patients had TD than PIGD, and Subtype III had the highest prevalence of PIGD. Compared with Subtype I and III, the second subtype had the highest TD prevalence but the lowest PIGD prevalence.

We also studied the longitudinal correlations between the described motor subtypes and our subtypes I-III. We observed that over time there was a larger cohort of patients transitioning from TD to PIGD for Subtype III compared with subtypes I-II. Over 6 years, the prevalence of PIGD in Subtype III increased from 20.8% to 48.7%, whereas for Subtype I, prevalence of PIGD increased from 18.9% at baseline to 32.3% after 6 years, and prevalence change in Subtype II was minimal, from 14.2% to 16.9% at 6 years. From the above comparisons, we can conclude that the three subtypes learned from our method had different compositions of the three known motor subtypes. According to a prior review^3^, PIGD PD often has poor prognosis with rapid progression while TD PD has a better prognosis with slower progression, which is consistent with the above progression analysis of Subtypes I, II, and III.

Similarly, we also investigated the correlations between the three learned subtypes and the three established cognitive subtypes: (1) no impairment (PD-NC), (2) mild impairment (PD-MCI), and dementia (PDD)^33–35^. Figure 6B demonstrates the results. For all three learned subtypes, the majority of patients were cognitively normal (PD-NC). Comparing with Subtype I and II, the prevalence of PD-MCI in Subtype III was the largest and increased significantly during the 6 years’ follow up. Moreover, Subtype III contained all PDD patients, and their prevalence increased from 0.64% to 7.79% over the 6-year horizon.

Finally, we computed the correlations between the three learned subtypes and mood subtypes. Specifically, we assessed four mood subtypes: (1) Anxiety; (2) Depression; (3) Depression-Anxiety; and (4) Normal. In PPMI, anxiety and depression were measured by STAI and GDS respectively, with higher scores indicating more severe anxiety or depression. A suggested cut-off point used for STAI is 54–55^36,37^. The cut-off point for GDS is 5 (patients with GDS $\geqslant$5 are “Depressed”; patients with GDS < 5 are “Not Depressed”). During the 6-year follow-up period, we observed that in Fig. 6C: for Subtype I, the prevalence of anxiety and mixed depression-anxiety decreased while the prevalence of depression alone increased; in contrast to Subtype I, the number of anxious patients slightly increased in Subtype II while the number of depression as well as mixed depression-anxiety patients decreased; in Subtype III, the number of patients with mixed depression-anxiety rose significantly, while the percentage of patients with anxiety and those with depression decreased, indicating a gradual transition from having one mood symptom to multiple mood symptoms (Fig. 6C). It is worth noticing that the prevalence of normal mood patients in all three learned subtypes slightly grew slightly in the follow-up period, suggesting that some patients had improvements in their mood disorder during the disease course.

In addition to the above mentioned conventional subtypes, a more traditional way for PD subtyping is just based on patient onset ages^38–40^. These studies suggested that PD patients with older onset ages are usually associated with more severe motor and non-motor symptoms^38,40^, and more rapid disease progression rates^39^. Our study takes a complimentary approach: we use longitudinal patient records for subtyping without onset ages. Table 1 shows average ages for the three subtypes. Of note Subtype III, the group with the most rapid progression, has the oldest average onset age of the three subtypes.

### Limitations

This study is an initial attempt on leveraging advanced data analytics for identification of PD subtypes with longitudinal and heterogeneous clinical study data. Our approach has demonstrated strong potentials of identification of comprehensive progressive PD subtypes. However, there are still some limitations in the current approach including (1) the approach is completely data-driven without utilization of any clinical domain knowledge; (2) the deep learning (LSTM) procedure cannot be straightforwardly interpreted; (3) our study is only conducted on the PPMI cohort. In the future, we will continue our research specifically on these lines, i.e., combining knowledge and data driven insights, making deep learning models interpretable, and replicate the findings on more patient cohorts.

While recognizing limitations of the present analyses based upon a single cohort in de novo PD patients, we suggest that the potential implications in the clinic are that individuals with milder PD motor and non-motor symptoms and lower CSF t-Tau levels will show moderate progression in motor and autonomic symptoms but are at lower risk of cognitive decline; those with mild motor symptoms but presence of significant cognitive deficits and anxiety, along with high CSF Abeta levels, are at risk of greater cognitive decline in the face of slow motor progression; and those with more severe motor combined with non-motor symptoms at onset are at risk of more rapid decline of motor and non-motor, including cognitive, symptoms. Our data therefore not only suggests dissociation of progression of motor versus cognitive symptom progression, but also dissociation between non-motor symptoms of cognition versus autonomic symptoms.

## Conclusions

A novel patient subtyping method for PD with deep learning model is proposed, where LSTM is leveraged to standardize and densify the patient records. After that, DTW is leveraged to calculate the patient similarities from which PD subtypes are derived. Using this novel approach we have identified three distinct subtypes in the PPMI cohort, demonstrating heterogeneous characteristics in both motor and non-motor characteristics. These subtypes have distinct patterns of progression, and moreover associate with specific biomarkers. We have examined how these newly discovered subtypes are related to traditional motor, cognitive and mood PD subtypes, and while we found some relationships we suggest that our approach benefits from incorporation of substantially more comprehensive data. The subtypes that we have identified demonstrate that, in contrast to studies that examine aggregate data, disease progression rates in our identified subtypes do not necessarily associate with baseline severity, and the progression rate of non-motor symptoms does not have a simple correlation with motor progression but varies by subtype.

## Supplementary information

Supplementary Material
