# Supplementary material for: Data-Driven Subtyping of Parkinson’s Disease Using Longitudinal Clinical Records: A Cohort Study
Source: Sci Rep. 2019 Jan 28;9:797. doi: 10.1038/s41598-018-37545-z (PMC6349906; doi:10.1038/s41598-018-37545-z)
Supplement: Supplementary file 1 — Supplementary Material [file 41598_2018_37545_MOESM1_ESM.pdf]

Supplementary Online Content

Data-Driven Subtyping of Parkinson's Disease Using Longitudinal Clinical Records: A Cohort Study

Xi Zhang, PhD[1], Jingyuan Chou, MS[1], Jian Liang, PhD[2], Cao Xiao, PhD[3], Yize Zhao, PhD[1], Harini Sarva, MD[4], Claire Henchcliffe, MD, DPhil[4], Fei Wang, PhD[1]

[1]Department of Healthcare Policy and Research. Weill Cornell Medical College, New York, NY, USA

[2]Department of Automation. Tsinghua University. Beijing. China.

[3]AI for Healthcare. IBM Research. Cambridge. MA. USA.

[4]Department of Neurology, Weill Cornell Medical College, New York, NY, USA

Corresponding Author:

Fei Wang, PhD
Department of Healthcare Policy and Research
Weill Cornell Medicine
425 East 61 Street, Suite 301
New York, NY 06032
Phone: +1 646 962 9405
few2001@med.cornell.edu

This supplementary material has been provided by the authors to give readers additional information about their work.

Table 1: The details of target variables

	Variable Name
**1**	Clinical Diagnosis
**2**	Demographics
**3**	Motor symptoms: MDS-UPDRS scores*
**4**	Cognitive Assessments: MoCA*
**5**	Cognitive Categorization: Normal Cognition; Mild Cognitive Impairment; Dementia
**6**	Other nonmotor variable: REM* Sleep Disorder
**7**	Biospecimen: Lumber Puncture Sample Collection
**8**	Biospecimen: Laboratory Procedures containing DNA, RNA, Urine, Plasma, & Serum samples
**9**	Imaging Results: DaTScan Striatal Binding Ratio
**10**	Imaging Results: Magnetic Resonance Imaging

* Abbreviations: MDS-UPDRS: Movement Disorders Society–revised Unified Parkinson's Disease Rating Scale; MoCA: Montreal Cognitive Assessment; REM: rapid eye movement

Table 2: Comparisons of p-values obtained by clustering different representation learning methods. The significant characteristics with p-value<.05 over 6-year follow-up as well as the progression during 6 years are marked by √

	Target Features Subtyping		All Features Subtyping		LSTM Representation Subtyping	
	6-Year Follow-up	Progression	6-Year Follow-up	Progression	6-Year Follow-up	Progression
Age onset	√		√		√	
Education			√			
H&Y Stage	√	√	√		√	√
MDS-UPDRS Part I	√	√		√	√	√
MDS-UPDRS Part II	√	√			√	√
MDS-UPDRS Part III	√		√			√
MDS-UPDRS Part IV				√		
MoCA	√	√			√	√
BJLO	√				√	√
ESS					√	
RBD	√			√	√	√
GDS	√	√		√	√	√
HVLT	√	√		√	√	√
LNS	√	√			√	√
SCOPA-AUT	√				√	
Semantic Fluency	√					√
STAI					√	
SDM	√				√	√
CSF Total tau					√	
CSF Abeta 42			√		√	
DaTScan Caudate	√	√			√	√
DaTScan Putamen	√	√			√	√
Medication Use	√	√	√	√	√	√

*Abbreviations: MDS-UPDRS: Movement Disorders Society–revised Unified Parkinson's Disease Rating Scale; MoCA: Montreal Cognitive Assessment; BJLO: Benton Judgment of Line Orientation; ESS: Epworth Sleepiness Scale; RBD: Rapid eye movement sleep Behavior Disorder; GDS: Geriatric Depression Scale; HVLT: Hopkin's Verbal Learning Test; LNS: Letter Number Sequencing; SCOPA-AUT: SCales for Outcomes

in PArkinson's disease-AUTomotic symptoms; STAI: State Trait Anxiety Inventory; SDM: Symbol Digit Modalities; CSF: Cerebrospinal fluid; DaTScan SBR: DaTScan Striatal Binding Ratio.

Table 3: Group Characteristics of patients at their 2nd year in the three subtypes

	Total	Subtype I	Subtype II	Subtype III	p-value
H&Y	1.77(0.50)	1.68 (0.48)	1.54 (0.55)	1.95 (0.48)	<0.0001[a], I vs III, II vs III
MDS-UPDRS Part I	6.84 (4.53)	5.43 (3.46)	5.25 (4.88)	9.05 (4.76)	<0.0001[a], I vs III, II vs III
MDS-UPDRS Part II	7.56 (4.95)	6.03 (3.74)	4.78 (3.63)	10.23 (5.37)	<0.0001[a], I vs III, II vs III
MDS-UPDRS Part III	24.61 (10.46)	21.56 (9.48)	21.2 (8.22)	29.33 (10.39)	0.0315[a], I vs III, II vs III
MDS-UPDRS Part IV	0.93 (1.90)	0.76 (1.70)	0.17 (0.37)	1.19 (2.15)	0.8854[b]
MoCA	26.14 (3.12)	27.17 (2.24)	27.33 (1.70)	24.95 (3.56)	0.4980[a]
BJLO	19.18 (6.99)	20.96 (7.00)	15.88 (5.72)	17.32 (6.47)	0.0491[a], I vs II, I vs III
ESS	6.48 (3.89)	5.71 (3.52)	3.75 (2.09)	6.64 (3.82)	0.0063[a], I vs III, II vs III
RBD[#]	3.30 (2.94)	2.45 (2.41)	2.00 (2.35)	4.86 (3.13)	0.0035[a], I vs III, II vs III
GDS	5.64 (1.73)	5.28 (1.36)	5.33 (1.65)	6.26 (2.06)	0.0016[a], I vs III
HVLT	24.05 (5.46)	26.41 (4.07)	24.5 (4.33)	20.29 (5.42)	<0.0001[a], I vs III, II vs III
LNS	9.84 (3.53)	11.02 (2.93)	8.33 (4.19)	8.35 (3.56)	0.0050[a], I vs II, I vs III
QUIP	0.13 (0.37)	0.12 (0.36)	0.08 (0.28)	0.16 (0.40)	0.9460[a]
SCOPA-AUT	12.47 (6.75)	10.17 (5.11)	8.83 (5.18)	16.67 (7.19)	0.0028[a], I vs III, II vs III
Semantic Fluency	50.09 (13.32)	53.89 (12.12)	49.58 (11.14)	44.28 (13.29)	0.3445[a]
STAI	64.61 (19.16)	61.34 (16.03)	56.83 (13.65)	70.62 (22.33)	0.5784[a]
SDMT	40.20 (10.85)	32.79 (11.08)	42.00 (5.46)	44.79 (8.13)	0.2780[a]
Medication Use[#]	2.01 (1.89)	2.31 (1.93)	0.29 (0.61)	2.05 (1.83)	<0.0001[a], I vs II, II vs III

[a]Chi-square test; [b]Fisher exact test. The specific different subtypes determined by use of Tukey post hoc analysis ($p<0.05$). Abbreviations: H&Y: Hoehn and Yahr; MDS-UPDRS: Movement Disorders Society–revised Unified Parkinson's Disease Rating Scale; MoCA: Montreal Cognitive Assessment; BJLO: Benton Judgment of Line Orientation; ESS: Epworth Sleepiness Scale; RBD: Rapid eye movement sleep Behavior Disorder; GDS: Geriatric Depression Scale; HVLT: Hopkin's Verbal Learning Test; LNS: Letter Number Sequencing; QUIP: Questionnaire for Impulsive-Compulsive Disorders in Parkinson's Disease; SCOPA-AUT: SCales for Outcomes in PArkinson's disease-AUTomotic symptoms; STAI: State Trait Anxiety Inventory; SDMT: Symbol Digit Modalities Test; MCI: Mild Cognitive Impairment. [#]RBD's rating scale is 0-10; MCI was determined by patients with cognititive declines, no functional impairment, and values of cognitive tests HVLT, BJLO, LNS, Semantic Fluency and SDMT; 1=normal, 2=Abnormal, not clinically significant, 3=Abnormal, clinically significant; Medication Use defined by 0=Unmedicated for PD, 1=Levadopa, 2=Dopamine Agonist, 3=Other, 4=Levadopa & Other, 5=Levadopa & Dopamine Agonist, 6=Dopamine Agonist & Other, 7=Levadopa & Dopamine Agonist & Other.

Table 4: Characteristics summarization of the learned subtypes

**Subtype I (43.1%)**	**Subtype II (22.9%)**	**Subtype III (33.9%)**
58.79 years at baseline	61.93 years at baseline	65.32 years at baseline
Mild motor symptoms at baseline	Moderate motor symptoms at baseline	Severe motor symptoms at baseline
Mild non-motor symptoms at baseline	Moderate non-motor symptoms at baseline	Severe non-motor symptoms at baseline
Moderate motor decline	Mild motor decline	Severe motor decline
Stable cognition, moderate RBD decline	Mild non-motor decline	Severe non-motor decline
Moderate DaTScan SBR decline	Mild DaTScan SBR decline	Severe DaTScan SBR decline

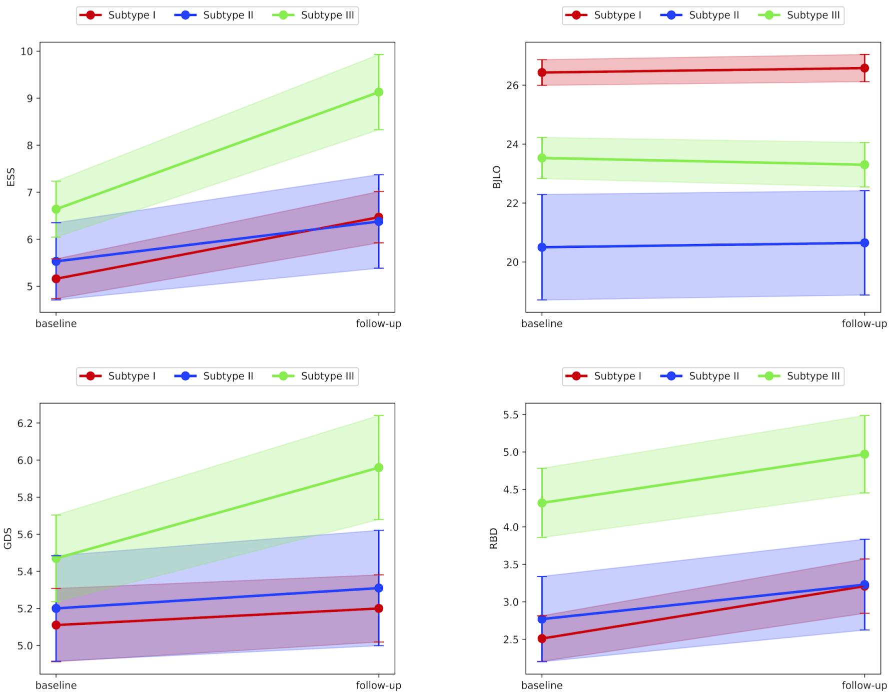

Figure 1: Comparisons of three subtypes on disease progression of the variables ESS, BJLO, GDS, and RBD. The time interval between baseline and follow-up is 6 years. The larger slope illustrates a more rapid progression on the corresponding variables. The representative variables with the p-value<0.05 are shown.

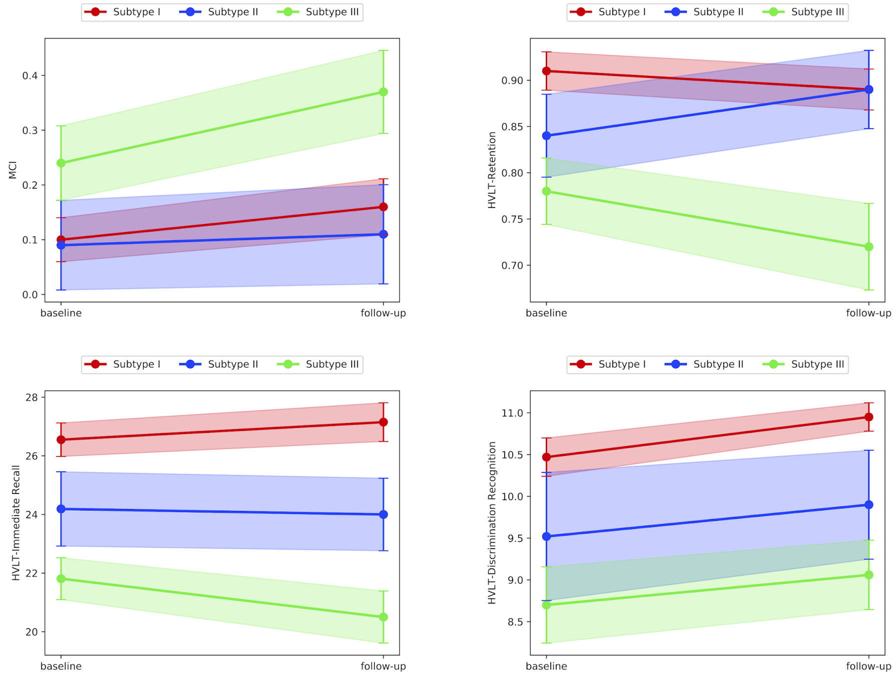

Figure 2: Comparisons of three subtypes on disease progression of the variables MCI and HVLT. The time interval between baseline and follow-up is 6 years. The larger slope illustrates a more rapid progression on the corresponding variables. The representative variables with the p-value<0.05 are shown.

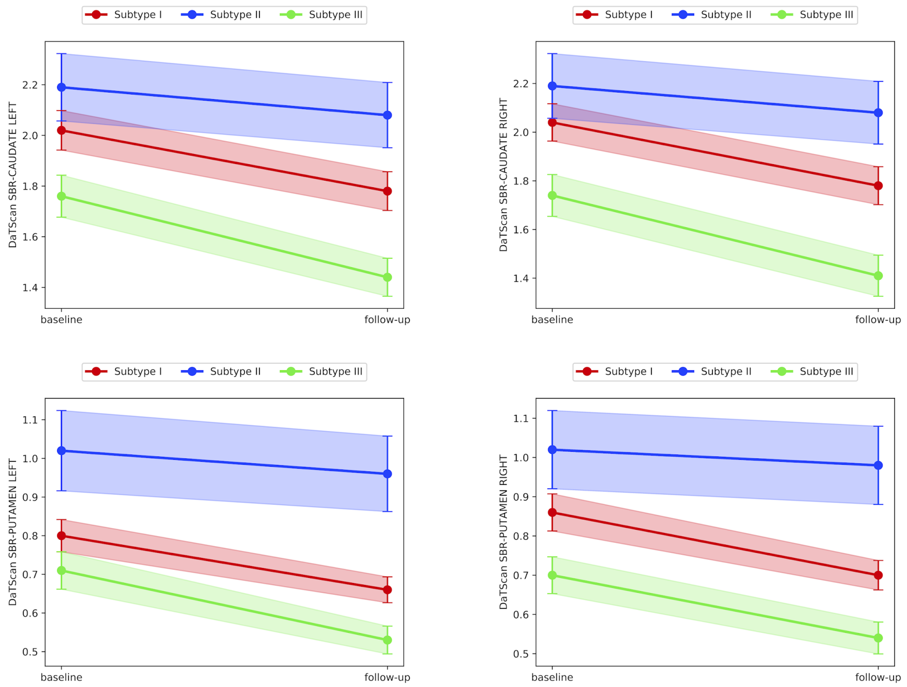

Figure 3: Comparisons of three subtypes on disease progression of the variables DaTScan Caudate and Putamen. The time interval between baseline and follow-up is 6 years. The larger slope illustrates a more rapid progression on the corresponding variables. The representative variables with the p-value<0.05 are shown.

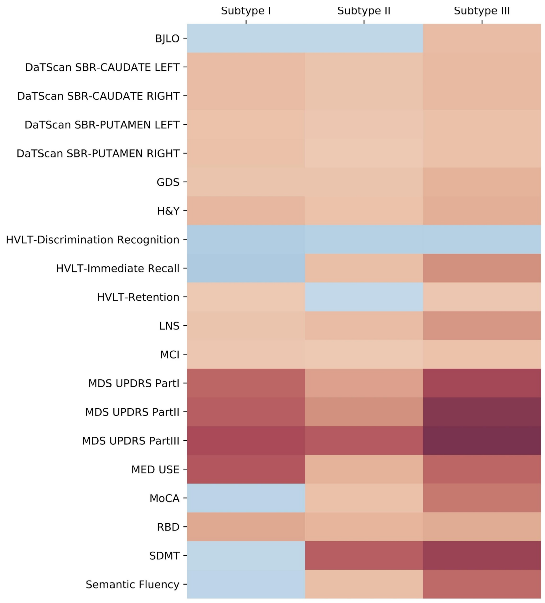

Figure 4: Heatmap illustration of the first-order difference of mean values for each subtype in LSTM results. It is obtained by the difference between the mean value of baseline and the mean value of the patients' last records on the variables. The red color represents a worse progression and the blue color shows a better progression on the symptoms of PD. The darker the color is, the significant the trend is. Variables with p-value<0.05 are shown.

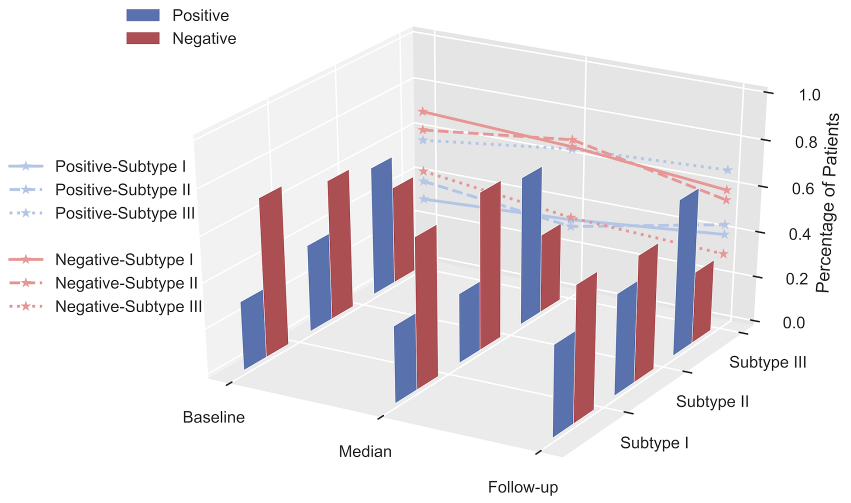

Figure 5: Patient correlation of three subtypes and RBD subtypes at baseline, median time point, and 6-year follow-up. Patients are categorized into RBD subtypes including Positive subtype, Negative subtype. The longitudinal correlation of three subtypes with Positive subtype and Negative subtype are plotted by lines respectively.

Table 5: Multivariate logistic regression model to find discriminant clinical predictors of Subtype I patients at baseline.

Age adjusted				Age unadjusted			
**variables**	**coefficie nt**	**95% CI**	**p-value**	**variables**	**coefficie nt**	**95% CI**	**p-value**
Age	-1.200	[-3.083, 0.635]	0.2039				
MDS-UPDRS Part I	-3.578	[-5.875, -1.406]	0.0016#	MDS-UPDRS Part I	-3.500	[-5.801, -1.325]	0.0021#
MDS-UPDRS Part II	0.113	[-2.015. 2.247]	0.9168	MDS-UPDRS Part II	0.165	[-1.950, 2.282]	0.8775
MDS-UPDRS Part III	-0.642	[-2.968, 1.637]	0.5823	MDS-UPDRS Part III	-0.547	[-2.871, 1.729]	0.6391
BJLO	4.327	[2.501, 6.339]	<0.0001#	BJLO	4.319	[2.497, 6.327]	<0.0001#
ESS	-1.592	[-3.449, 0.222]	0.0880	ESS	-1.560	[-3.410, 0.249]	0.0935
GDS	-0.526	[-2.550, 1.485]	0.6075	GDS	-0.335	[-2.315, 1.642]	0.7387
HVLT	5.057	[3.160, 7.087]	<0.0001#	HVLT	5.202	[3.325, 7.216]	<0.0001#
LNS	0.185	[-1.923, 2.323]	0.8639	LNS	0.463	[-1.597, 2.559]	0.6609
MoCA	-0.517	[-2.328, 1.281]	0.5725	MoCA	-0.501	[-2.310, 1.295]	0.5843
QUIP	0.028	[-1.509, 1.534]	0.9701	QUIP	0.045	[-1.485, 1.542]	0.9532
RBD	-0.969	[-2.281, 0.322]	0.1427	RBD	-0.884	[-2.187, 0.400]	0.1788
SCOPA-AUT	-1.611	[-4.055, 0.744]	0.1868	SCOPA-AUT	-2.052	[-4.407, 0.209]	0.0805
Semantic Fluency	2.227	[-0.349, 4.840]	0.0918	Semantic Fluency	2.288	[-0.267, 4.882]	0.0809
STAI	-0.528	[-2.354, 1.292]	0.5688	STAI	-0.315	[-2.091, 1.465]	0.7270
**SDM**	**2.536**	**[-0.247, 5.449]**	**0.0799**	**SDM**	**3.123**	**[0.479, 5.907]**	**0.0237#**
CAUDATE.RIGHT*	-0.713	[-4.314, 2.877]	0.6963	CAUDATE.RIGHT*	-0.482	[-4.067, 3.095]	0.7911

CAUDATE.LEFT*	3.173	[-0.314, 6.734]	0.0766	CAUDATE.LEFT*	3.022	[-0.449, 6.557]	0.0897
PUTAMEN.RIGHT*	1.329	[-1.739, 4.447]	0.3982	PUTAMEN.RIGHT*	1.171	[-1.872, 4.258]	0.4527
PUTAMEN.LEFT*	-4.076	[-7.603.-0.563]	0.0222#	PUTAMEN.LEFT*	-4.016	[-7.535, -0.501]	0.0241#
Duration	-1.131	[-2.666, 0.387]	0.1442	Duration	-1.201	[-2.737, 0.317]	0.1210
Education	-1.590	[-3.825, 0.597]	0.1571	Education	-1.771	[-3.985, 0.390]	0.1111
H&Y	0.301	[-0.386, 1.006]	0.3947	HY	0.255	[-0.426, 0.953]	0.4661
MCI	0.049	[-0.780, 0.873]	0.9055	MCI	0.021	[-0.807, 0.844]	0.9584
Gender	-0.074	[-0.739, 0.585]	0.8240	Gender	-0.055	[-0.721, 0.605]	0.8696

Statistical significant correlation (p-value<0.05); * LEFT/RIGH means left/right Caudate or Putamen; Bold variables are p-values changed from significant (not significant) to not significant (significant), with/without Age adjustment.

Table 6: Multivariate logistic regression model to find discriminant clinical predictors of Subtype II patients at baseline.

Age adjusted				Age unadjusted			
**variables**	**coefficient**	**95% CI**	**p-value**	**variables**	**coefficient**	**95% CI**	**p-value**
Age	0.680	[-1.889, 3.340]	0.6075				
MDS-UPDRS Part I	3.941	[0.905, 7.050]	0.0112#	MDS-UPDRS Part I	3.851	[0.829, 6.962]	0.0130#
MDS-UPDRS Part II	-3.886	[-7.620,-0.613]	0.0281#	MDS-UPDRS Part II	-3.846	[-7.589, -0.574]	0.0300#
MDS-UPDRS Part III	-0.414	[-4.020, 3.078]	0.8181	MDS-UPDRS Part III	-0.419	[-4.018, 3.065]	0.8153
BJLO	-3.508	[-5.680,-1.413]	0.0011#	BJLO	-3.547	[-5.721, -1.452]	0.0010#
ESS	1.196	[-1.169, 3.552]	0.3162	ESS	1.163	[-1.198, 3.512]	0.3288
GDS	-0.093	[-2.991, 2.716]	0.9484	GDS	-0.213	[-3.080, 2.573]	0.8814
HVLT	-1.869	[-4.429, 0.623]	0.1447	HVLT	-1.964	[-4.504, -0.510]	0.1225
LNS	-1.489	[-4.413, 1.313]	0.3053	LNS	-1.673	[-4.516, 1.068]	0.2377
MoCA	1.107	[-1.120, 3.473]	0.3410	MoCA	1.175	[-1.028, 3.524]	0.3082
QUIP	1.433	[-0.756, 3.358]	0.1623	QUIP	1.441	[-0.746, 3.363]	0.1595
RBD	-1.266	[-3.456, 0.759]	0.2363	RBD	-1.303	[-3.482, 0.708]	0.2200
SCOPA-AUT	-3.826	[-7.696, -0.349]	0.0396#	SCOPA-AUT	-3.604	[-7.407, -0.235]	0.0470#
Semantic Fluency	0.555	[-3.000, 3.962]	0.7531	Semantic Fluency	0.527	[-3.020, 3.925]	0.7648
STAI	-4.205	[-7.213, -1.441]	0.0041#	STAI	-4.289	[-7.278, -1.552]	0.0031#
SDM	3.054	[-0.825, 6.934]	0.1204	SDM	2.649	[-0.900, 6.249]	0.1440
CAUDATE.RIGHT*	5.915	[1.191, 10.890]	0.0160#	CAUDATE.RIGHT*	5.744	[1.078, 10.639]	0.0177#
CAUDATE.LEFT*	-6.080	[-10.716,-1.639]	0.0082#	CAUDATE.LEFT*	-6.016	[-10.629,-1.599]	0.0085#
PUTAMEN.RIGHT*	-0.156	[-3.945, 3.500]	0.9340	PUTAMEN.RIGHT*	-0.059	[-3.811, 3.570]	0.9746
PUTAMEN.LEFT*	9.115	[5.057, 13.562]	<0.0001#	PUTAMEN.LEFT*	9.115	[5.069, 13.547]	<0.0001#
Duration	1.538	[-0.521, 3.500]	0.1298	Duration	1.581	[-0.460, 3.533]	0.1168
Education	1.160	[-1.704, 4.082]	0.4287	Education	1.328	[-1.479, 4.182]	0.3546
HY	-0.584	[-1.616, 0.406]	0.2542	HY	-0.541	[-1.556, 0.435]	0.2835
MCI	-0.040	[-1.555, 1.279]	0.9545	MCI	-0.019	[-1.517, 1.291]	0.9782

Gender	0.417	[-0.450, 1.320]	0.3518	Gender	0.404	[-0.463, 1.306]	0.3681

[#] Statistical significant correlation (p-value<0.05); [*] LEFT/RIGH means left/right Caudate or Putamen.

Table 7: Multivariate logistic regression model to find discriminant clinical predictors of Subtype III patients at baseline.

Age adjusted				Age unadjusted			
**variables**	**coefficient**	**95% CI**	**p-value**	**variables**	**coefficient**	**95% CI**	**p-value**
Age	1.465	[-0.745,3.749]	0.1995				
MDS-UPDRS Part I	2.696	[0.349,5.109]	0.0256[#]	MDS-UPDRS Part I	0.119	[0.013, 0.229]	0.0286[#]
MDS-UPDRS Part II	0.752	[-1.488,3.021]	0.5101	MDS-UPDRS Part II	0.033	[-0.058,0.128]	0.4734
MDS-UPDRS Part III	1.573	[-1.002,4.261]	0.2395	MDS-UPDRS Part III	0.026	[-0.020, 0.755]	0.2745
BJLO	-2.457	[-4.448,-0.513]	0.0138[#]	BJLO	-0.107	[-0.192,-0.024]	0.0118[#]
ESS	1.037	[-0.948,3.078]	0.3108	ESS	0.047	[-0.051,0.149]	0.3462
GDS	0.549	[-1.788,2.905]	0.6449	GDS	0.042	[-0.188, 0.274]	0.7176
HVLT	-4.768	[-7.053,-2.624]	<0.0001[#]	HVLT	-0.182	[-0.266, -0.103]	<0.0001[#]
LNS	0.338	[-2.159,2.835]	0.7895	LNS	-0.001	[-0.143, 0.141]	0.9900
MoCA	-0.141	[-2.200,1.908]	0.8925	MoCA	-0.009	[-0.167, 0.147]	0.9039
QUIP	-0.510	[-2.280,1.238]	0.5668	QUIP	-0.247	[-1.127, 0.624]	0.5778
RBD	1.352	[-0.039, 2.767]	0.0580	RBD	0.111	[-0.014,0.239]	0.0828
SCOPA-AUT	3.891	[1.257,6.703]	0.0049[#]	SCOPA-AUT	0.109	[0.046,0.177]	0.0011[#]
SDM	-4.766	[-8.302,-1.471]	0.0060[#]	SDM	-0.072	[-0.118,-0.030]	0.0013[#]
Semantic Fluency	-2.726	[-5.946,0.418]	0.0921	Semantic Fluency	-0.034	[-0.073, 0.003]	0.0736
STAI	2.870	[0.773,5.053]	0.0082[#]	STAI	0.026	[0.006, 0.048]	0.0144[#]
CAUDATE.LEFT[*]	1.188	[-2.903,5.332]	0.5699	CAUDATE.LEFT[*]	0.397	[-0.824,1.636]	0.5249
CAUDATE.RIGHT[*]	-2.229	[-6.357,1.820]	0.2830	CAUDATE.RIGHT[*]	-0.707	[-1.860,0.425]	0.2228
PUTAMEN.LEFT[*]	-4.510	[-8.870,-0.240]	0.0397[#]	PUTAMEN.LEFT[*]	-1.799	[-3.540, -0.096]	0.0396[#]
PUTAMEN.RIGHT[*]	-3.037	[-6.876, 0.654]	0.1121	PUTAMEN.RIGHT[*]	-1.200	[-2.787, 0.328]	0.1292
Duration	0.575	[-1.247, 2.353]	0.5279	Duration	0.018	[-0.030, 0.067]	0.4381
Education	0.796	[-1.680,3.280]	0.5271	Education	0.049	[-0.066, 0.166]	0.4034
HY	-0.048	[-0.844,0.734]	0.9034	HY	-0.012	[-0.802, 0.766]	0.9762
MCI	-0.050	[-0.911,0.808]	0.9070	MCI	-0.032	[-0.893, 0.829]	0.9417
Gender	-0.258	[-1.013,0.491]	0.4986	Gender	-0.283	[-1.033, 0.462]	0.4568

[#] Statistical significant correlation (p-value<0.05); [*] LEFT/RIGH means left/right Caudate or Putamen.

Table 8: Multivariate logistic regression model to find discriminant clinical predictors of Subtype I patients at last records.

Age adjusted				Age unadjusted			
**variables**	**coefficient**	**95% CI**	**p-value**	**variables**	**coefficient**	**95% CI**	**p-value**

Age	0.009	[-1.867, 1.904]	0.9920				
MDS-UPDRS Part I	-2.239	[-4.826, 0.255]	0.0832	MDS-UPDRS Part I	-2.239	[-4.826, 0.253]	0.0832
MDS-UPDRS Part II	-2.082	[-4.976, 0.747]	0.1521	MDS-UPDRS Part II	-2.083	[-4.968, 0.736]	0.1506
MDS-UPDRS Part III	-1.629	[-3.834, 0.495]	0.1388	MDS-UPDRS Part III	-1.629	[-3.831, 0.493]	0.1385
BJLO	2.098	[0.094, 4.187]	0.0436[#]	BJLO	2.097	[0.097, 4.184]	0.0434[#]
ESS	-1.426	[-3.337, 0.474]	0.1404	ESS	-1.427	[-3.330, 0.465]	0.1384
GDS	-1.971	[-4.469, 0.491]	0.1173	GDS	-1.973	[-4.448, 0.470]	0.1138
HVLT	3.900	[1.840, 6.046]	0.0002[#]	HVLT	3.899	[1.841, 6.045]	0.0002[#]
LNS	1.679	[-0.907, 4.385]	0.2117	LNS	1.677	[-0.885, 4.349]	0.2074
MoCA	2.745	[-0.697, 6.305]	0.1228	MoCA	2.744	[-0.696, 6.299]	0.1225
QUIP	0.475	[-1.347, 2.328]	0.6084	QUIP	0.475	[-1.346, 2.328]	0.6082
**RBD**	**-1.424**	**[-2.882,-0.005]**	**0.0514**	**RBD**	**-1.424**	**[-2.872,-0.016]**	**0.0496[#]**
SCOPA-AUT	-0.472	[-2.936, 1.946]	0.7032	SCOPA-AUT	-0.469	[-2.862, 1.874]	0.6964
Semantic Fluency	2.857	[0.347, 5.478]	0.0284[#]	Semantic Fluency	2.857	[0.349, 5.476]	0.0283[#]
STAI	-1.022	[-2.926, 0.873]	0.2895	STAI	-1.023	[-2.915, 0.859]	0.2858
SDM	4.399	[0.462, 8.463]	0.0306[#]	SDM	4.393	[0.630, 8.282]	0.0239[#]
CAUDATE.RIGHT[*]	0.425	[-3.119, 3.992]	0.8138	CAUDATE.RIGHT[*]	0.423	[-3.099, 3.970]	0.8136
CAUDATE.LEFT[*]	3.417	[-0.439, 7.367]	0.0849	CAUDATE.LEFT[*]	3.418	[-0.436, 7.365]	0.0847
PUTAMEN.RIGHT[*]	-1.061	[-4.921, 2.739]	0.5864	PUTAMEN.RIGHT[*]	-1.059	[-4.892, 2.711]	0.5844
PUTAMEN.LEFT[*]	-4.386	[-8.295, -0.594]	0.0249[#]	PUTAMEN.LEFT[*]	-4.387	[-8.291, -0.599]	0.0247[#]
Duration	-0.863	[-2.548, 0.851]	0.3168	Duration	-0.863	[-2.546, 0.851]	0.3167
Education	-0.486	[-2.819, 1.877]	0.6836	Education	-0.485	[-2.811, 1.871]	0.6833
HY	0.029	[-0.585, 0.648]	0.9244	HY	0.029	[-0.583, 0.647]	0.9236
MCI	0.513	[-0.247, 1.298]	0.1907	MCI	0.514	[-0.246, 1.298]	0.1900
Gender	0.502	[-0.221, 1.239]	0.1761	Gender	0.502	[-0.221, 1.239]	0.1761
MED-USE[a]	2.296	[1.263, 3.393]	<0.0001[#]	MED-USE	2.295	[1.272, 3.384]	<0.0001[#]

[#] Statistical significant correlation (p-value<0.05); [*] LEFT/RIGH means left/right Caudate or Putamen; Bold variables are p-values changed from significant (not significant) to not significant (significant), with/without Age adjustment; [a] Medication Use defined by 0=Unmedicated for PD, 1=Levadopa, 2=Dopamine Agonist, 3=Other, 4=Levadopa & Other, 5=Levadopa & Dopamine Agonist, 6=Dopamine Agonist & Other, 7=Levadopa & Dopamine Agonist & Other.

Table 9: Multivariate logistic regression model to find discriminant clinical predictors of Subtype II patients at last records.

Age adjusted				Age unadjusted			
**variables**	**coefficient**	**95% CI**	**p-value**	**variables**	**coefficient**	**95% CI**	**p-value**
Age	0.326	[-2.237, 2.928]	0.8031				
MDS-UPDRS Part	0.903	[-2.918, 4.618]	0.6335	MDS-UPDRS Part	0.938	[-2.871, 4.644]	0.6197

I				I			
MDS-UPDRS Part II	-2.043	[-6.874, 2.365]	0.3823	MDS-UPDRS Part II	-2.021	[-6.83, 2.374]	0.3858
MDS-UPDRS Part III	1.493	[-1.868, 4.826]	0.3766	MDS-UPDRS Part III	1.450	[-1.89, 4.745]	0.3861
BJLO	-2.333	[-4.882, 0.142]	0.0654	BJLO	-2.350	[-4.899, 0.129]	0.0638
ESS	-0.192	[-2.969, 2.502]	0.8894	ESS	-0.227	[-2.990, 2.451]	0.8689
GDS	0.469	[-3.396, 4.337]	0.8106	GDS	0.424	[-3.424, 4.268]	0.8276
HVLT	-5.245	[-9.095, -1.683]	0.0051[#]	HVLT	-5.224	[-9.049, -1.676]	0.0051[#]
LNS	-0.434	[-4.569, 3.605]	0.8334	LNS	-0.468	[-4.591, 3.564]	0.8203
MoCA	4.765	[-0.143, 10.113]	0.0668	MoCA	4.769	[-0.129, 10.107]	0.0661
QUIP	2.095	[-0.693, 4.571]	0.1097	QUIP	2.110	[-0.660, 4.574]	0.1052
RBD	-0.236	[-2.598, 1.996]	0.8384	RBD	-0.275	[-2.618, 1.930]	0.8105
SCOPA-AUT	-4.916	[-9.330, -0.890]	0.0212[#]	SCOPA-AUT	-4.792	[-9.076, -0.879]	0.0206[#]
Semantic Fluency	1.673	[-2.518, 5.807]	0.4270	Semantic Fluency	1.579	[-2.546, 5.648]	0.4462
STAI	-2.192	[-5.590, 0.739]	0.1714	STAI	-2.224	[-5.611, 0.693]	0.1634
SDM	-1.103	[-7.329, 4.919]	0.7244	SDM	-1.251	[-7.375, 4.634]	0.6832
CAUDATE.RIGHT[*]	4.259	[-0.962, 9.636]	0.1121	CAUDATE.RIGHT[*]	4.171	[-1.004, 9.488]	0.1160
CAUDATE.LEFT[*]	-6.522	[-12.918,-0.438]	0.0390[#]	CAUDATE.LEFT[*]	-6.474	[-12.867, -0.397]	0.0403[#]
PUTAMEN.RIGHT[*]	1.913	[-2.967, 6.943]	0.4459	PUTAMEN.RIGHT[*]	2.015	[-2.774, 6.984]	0.4147
PUTAMEN.LEFT[*]	9.991	[4.621, 15.938]	0.0005[#]	PUTAMEN.LEFT[*]	9.940	[4.589, 15.874]	0.0005[#]
Duration	1.805	[-0.441, 3.999]	0.1066	Duration	1.816	[-0.423, 4.004]	0.1037
Education	0.601	[-2.807, 4.019]	0.7277	Education	0.684	[-2.646, 4.041]	0.6862
HY	-0.838	[-1.827, 0.110]	0.0869	HY	-0.838	[-1.828, 0.111]	0.0871
MCI	-0.735	[-2.140, 0.504]	0.2686	MCI	-0.719	[-2.117, 0.512]	0.2760
Gender	-0.026	[-1.100, 1.062]	0.9614	Gender	-0.038	[-1.108, 1.046]	0.9436
MED-USE[a]	-4.609	[-6.939, -2.677]	<0.0001[#]	MED-USE	-4.646	[-6.959, -2.725]	<0.0001[#]

[#] Statistical significant correlation (p-value<0.05); [*] LEFT/RIGH means left/right Caudate or Putamen; [a] Medication Use defined by 0=Unmedicated for PD, 1=Levadopa, 2=Dopamine Agonist, 3=Other, 4=Levadopa & Other, 5=Levadopa & Dopamine Agonist, 6=Dopamine Agonist & Other, 7=Levadopa & Dopamine Agonist & Other.

Table 10: Multivariate logistic regression model to find discriminant clinical predictors of Subtype III patients at last records.

Age adjusted				Age unadjusted			
variables	coefficient	95% CI	p-value	variables	coefficient	95% CI	p-value
Age	2.246	[-0.146, 4.746]	0.0703				
MDS-UPDRS Part I	1.795	[-0.998, 4.662]	0.2113	MDS-UPDRS Part I	1.725	[-1.100, 4.620]	0.2353
MDS-UPDRS Part II	3.421	[0.253, 6.762]	0.0381[#]	MDS-UPDRS Part II	3.223	[0.084, 6.512]	0.0480[#]

MDS-UPDRS Part III	1.477	[-0.907, 3.964]	0.2318	MDS-UPDRS Part III	1.492	[-0.912, 4.001]	0.2309
BJLO	-0.368	[-2.642, 1.966]	0.7531	BJLO	-0.463	[-2.727, 1.866]	0.6915
ESS	2.299	[0.006, 4.649]	0.0508	ESS	2.000	[-0.243, 4.283]	0.0814
GDS	0.922	[-1.969, 3.861]	0.5342	GDS	0.756	[-2.086, 3.621]	0.6025
HVLT	-2.821	[-5.332, -0.394]	0.0244#	HVLT	-3.032	[-5.521, -0.635]	0.0144#
LNS	-1.828	[-5.051, 1.263]	0.2540	LNS	-2.165	[-5.353, 0.897]	0.1724
MoCA	-4.826	[-8.710, -1.162]	0.0117#	MoCA	-4.540	[-8.363, -0.915]	0.0163#
QUIP	-1.524	[-3.734, 0.632]	0.1673	QUIP	-1.462	[-3.655, 0.678]	0.1809
RBD	1.576	[0.023, 3.187]	0.0496#	RBD	1.338	[-0.184, 2.906]	0.0882
SCOPA-AUT	3.637	[0.637, 6.827]	0.0205#	SCOPA-AUT	4.119	[1.219, 7.229]	0.0069#
SF	-5.021	[-8.295, -1.944]	0.0018#	SF	-4.956	[-8.173, -1.931]	0.0017#
STAI	2.529	[0.384, 4.740]	0.0219#	STAI	2.179	[0.077, 4.344]	0.0437#
SDM	-3.197	[-7.855, 1.345]	0.1714	SDM	-4.251	[-8.765, 0.134]	0.0600
CAUDATE.RIGHT*	-0.081	[-3.974, 3.788]	0.9668	CAUDATE.RIGHT*	-0.256	[-4.087, 3.561]	0.8947
CAUDATE.LEFT*	0.086	[-4.458, 4.651]	0.9700	CAUDATE.LEFT*	-0.059	[-4.565, 4.462]	0.9795
**PUTAMEN.RIGHT***	**-5.313**	**[-10.397,-0.51]**	**0.0343#**	**PUTAMEN.RIGHT***	**-4.776**	**[-9.784, -0.048]**	**0.0535**
PUTAMEN.LEFT*	-2.657	[-8.128, 2.700]	0.3355	PUTAMEN.LEFT*	-2.659	**[-8.096, 2.629]**	0.3294
Duration	0.194	[-1.914, 2.253]	0.8544	Duration	0.136	[-1.976, 2.197]	0.8978ß
Education	-0.947	[-4.014, 2.024]	0.5372	Education	-0.621	[-3.637, 2.299]	0.6808
HY	0.261	[-0.492, 1.021]	0.4955	HY	0.352	[-0.392, 1.105]	0.3542
MCI	-0.038	[-0.881, 0.794]	0.9285	MCI	-0.017	[-0.860, 0.814]	0.9673
Gender	-0.945	[-1.821, -0.103]	0.0302#	Gender	-0.849	[-1.708, -0.020]	0.0474#
MED-USE[a]	0.286	[-0.956, 1.552]	0.6527	MED-USE[a]	-0.007	[-1.202, 1.195]	0.9907

[#] Statistical significant correlation (p-value<0.05); [*] LEFT/RIGH means left/right Caudate or Putamen; Bold variables are p-values changed from significant (not significant) to not significant (significant), with/without Age adjustment; [a] Medication Use defined by 0=Unmedicated for PD, 1=Levadopa, 2=Dopamine Agonist, 3=Other, 4=Levadopa & Other, 5=Levadopa & Dopamine Agonist, 6=Dopamine Agonist & Other, 7=Levadopa & Dopamine Agonist & Other.

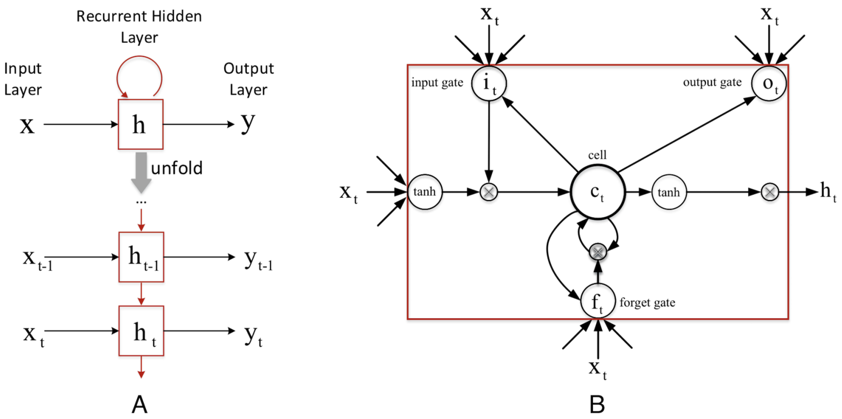

Figure 6: The LSTM recurrent neural network. (A) the simple recurrent neural network architecture. (B) long short-term memory cell.

Figure 4 shows the architecture of LSTM. The input vector at time step t of the pth patient can be denoted as $x_t \in R^d, t = 1, \ldots, N_p$, where the number of unique record timestamps for the patient is N_p and d is the dimensionality input feature. The number of total records provided for the model is an aggregation of patient records $N = \sum_p N_p$. Each patient may have a different length of record sequences We subsequently introduce a memory cell, which is employed in hidden layer h_t at timestamp t. We used a simplified version of the memory unit in Figure 4B. Mathematically, it is implemented by the following composite functions:

$$i_t = \sigma(W_i x_t + W_i h_{t-1} + b_i)$$

$$f_t = \sigma\left(W_f x_t + W_f h_{t-1} + b_f\right)$$

$$o_t = \sigma(W_o x_t + W_o h_{t-1} + b_o)$$

$$c_t = f_t .* c_{t-1} + i_t .* tanh(W_c x_t + W_c h_{t-1} + b_c)$$

$$h_t = o_t .* tanh\,(c_t)$$

where $\sigma(x) = 1/(1 + exp\,(-x))$ is the logistic sigmoid function, i, f, o and c are the input gate, forget gate, output gate, and cell state, respectively. The vector $h_t \in R^k, k \ll d$ is a compact continuous low-dimensional embedding for each input x_t. There are two types of target features: binary and continuous. We construct two different types of losses to measure the prediction performance at each time stamp. Specifically, for each timestamp t, the loss on binary dimension y_t^b is measured by the following penalized logistic loss:

$$\sum_t \sum_{j=1}^{m_b} log\,(1 + exp\,(-y_{t,j}^b (w_{b,j}^T h_t))) + \lambda \|W_b\|_F^2$$

where j indicates the dimension of binary target value. For continuous targets, y_t^g ,the loss is measured by the following penalized square loss:

$$\frac{1}{2} \sum_t \|y_t^g - W_g h_t\|_2^2 + \lambda \|W_g\|_F^2$$

where y_t^g is the continuous part of y_t. $\|\cdot\|_F$ is Frobenius norm. In both loss functions, λ is a hyperparameter to control the contribution of regularizers. The target $y_t \in R^m$ consists of the binary part y_t^b and continuous part y_t^g. In total, the parameter collection $\{W_i, b_i, W_f, b_f, W_o, b_o, W_c, b_c, W_g, W_b\}$ can be optimized jointly through back-propagation and mini-batch stochastic gradient descent. We implemented the algorithm using MATLAB software.
